# The influence of an environmentally relevant polychlorinated biphenyl mixture on the intestinal microbiota in post-weaning mouse dams

**DOI:** 10.1007/s11356-026-37418-3

**Published:** 2026-01-22

**Authors:** Hui Wang, Laura E. Dean, Xueshu Li, Rachel L. Fitzjerrells, Kai Wang, Ashutosh K. Mangalam, Rachel F. Marek, Conner L. Kennedy, Monica M. Ridlon, Audrey Spiegelhoff, Kimberly P. Keil Stietz, Hans-Joachim Lehmler

**Affiliations:** 1 Department of Occupational and Environmental Health, The University of Iowa, Iowa City, IA USA; 2 Interdisciplinary Graduate Program in Informatics, The University of Iowa, Iowa City, IA USA; 3 College of Dentistry, The University of Iowa, Iowa City, IA USA; 4 Department of Biostatistics, The University of Iowa, Iowa City, IA USA; 5 Department of Pathology, The University of Iowa, Iowa City, IA USA; 6 Department of Civil and Environmental Engineering, The University of Iowa, Iowa City, IA USA; 7 Department of Comparative Biosciences, University of Wisconsin-Madison, Madison, WI USA

**Keywords:** Environmental contaminants, Environmental stress, PCBs, Metabolism, Intestinal microbiota, Mice, Multi-omics integration

## Abstract

Polychlorinated biphenyls (PCBs) are environmental pollutants linked to neurological impairments by mechanisms involving the gut microbiome. This study examines the effects of maternal exposure to a PCB mixture on the gut microbiome of post-weaning mouse dams. Female C57BL/6 J mice were exposed daily to PCBs at different doses (0, 0.1, 1, or 6 mg/kg body weight/day) before mating, and throughout gestation and lactation. Post-weaning intestinal contents from dams were analyzed to assess microbiome abundance and quantify PCBs and their metabolites. PCB exposure affected the composition of microbial taxa, though not significantly. However, topic modeling analysis identified a distinct microbial community that was significantly more prevalent in the control group compared to post-weaning mouse dams exposed to the MARBLES mixture at medium and high doses. Complex PCBs and metabolite mixtures were detected in the intestinal contents. Certain PCBs and their metabolites were associated with changes in bacterial abundance, as determined by network analysis. These findings show that individual PCBs and their metabolites can affect gut microbial communities, potentially contributing to neurological impairments in mice exposed to PCBs during gestation and lactation.

**Graphical Abstract:**

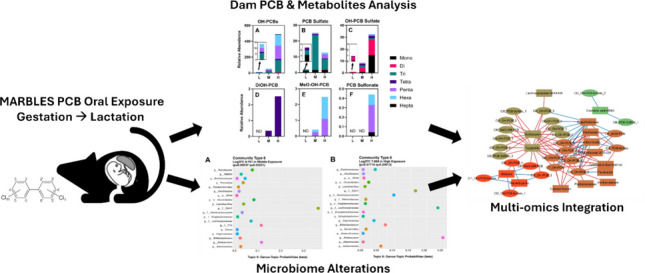

**Supplementary Information:**

The online version contains supplementary material available at 10.1007/s11356-026-37418-3.

## Introduction

Polychlorinated biphenyls (PCBs) are harmful environmental pollutants comprising 209 unique chemical compounds, distinguished by the number and position of chlorine substituents (IARC 2015). Each unique PCB is known as a congener. PCBs are non-flammable, chemically stable, and have electrical insulating properties (Erickson and Kaley 2011). As a result, they were widely used in various applications, including electrical transformers, capacitors, caulking, adhesives, and hydraulic systems, starting in the 1920 s (Erickson and Kaley 2011). Due to their environmental persistence and adverse health effects, the EPA banned their production in the United States in 1979, followed by global restrictions under the Stockholm Convention on Persistent Organic Pollutants (Lallas 2001; Erickson and Kaley 2011).

Despite these bans, PCBs remain prevalent in the environment, leaching from contaminated dump sites, deteriorating equipment, and building materials (ATSDR 2000; IARC 2015). Additionally, new sources of PCB contamination have emerged, particularly as byproducts in paint pigment manufacturing, raising continuous public health concerns (Jahnke and Hornbuckle 2019). The EPA has set a maximum contaminant level of 0.0005 ppm in public drinking water (EPA 2009), and the FDA mandates tolerance levels of 0.2–3.0 ppm for all foods, and 10 ppm in paper food packaging (FDA 1998). Numerous studies have shown strong links between PCB exposure and adverse health effects, including neurological impairments, endocrine disruption, immune suppression, and reproductive and developmental disorders (Kramer et al. 2012; Bell 2014; Peixoto-Rodrigues et al. 2025). The International Agency for Research on Cancer (IARC) classifies PCBs as Group 1 carcinogens, indicating that they are carcinogenic to humans based on sufficient evidence from human studies, animal experiments, and supporting mechanistic data (IARC 2015).

Besides cancer outcomes, the developmental neurotoxicity of PCBs represents a significant adverse health outcome associated with PCB exposure. The “Markers of Autism Risk in Babies: Learning Early Signs” (MARBLES) is a prospective cohort study focused on identifying early risks of autism spectrum disorders in infants, including exposure to PCBs (Hertz-Picciotto et al. 2018). This study enrolls women who are pregnant or planning to become pregnant in Northern California and are at an elevated risk of having another child with an autism spectrum disorder. The 12 most common PCB congeners have been identified in the serum of this at-risk population (Sethi et al. 2019). The congener profile observed in this cohort differs from PCB profiles historically reported in the general United States human populations (Patterson et al. 2009), with lower-chlorinated congeners such as PCB 28 contributing a substantial proportion of the total serum PCB burden. This profile likely reflects background-level exposures that could be a combination of contemporary and legacy PCB sources. A synthetic mixture of PCBs, known as the MARBLES mix, was formulated to replicate the PCB profiles measured in this cohort (Sethi et al. 2019). Developmental exposure to this mixture in utero and during lactation has been demonstrated to disrupt social and repetitive behaviors in juvenile mice in a sex- and genotype-dependent manner (Granillo et al. 2019; Keil Stietz et al. 2021). However, the mechanisms by which PCBs impact the post-weaning dam remain unknown.

The gut microbiome, a community of microorganisms including bacteria and fungi residing in the gut (Freedman et al. 2018), is increasingly recognized as essential for human health. It contributes to gastrointestinal balance, metabolic health, immune regulation, and neurological processes (Mayer 2011; Dupont et al. 2020). These effects are mediated in part through the gut-liver-brain axis, a complex communication network that integrates metabolic, neural, and immune signaling between the gut microbiota, liver, and brain (Giovannini et al. 2021; Afzaal et al. 2022; Yan et al. 2023). The pathophysiology of psychiatric disorders, including neurodevelopmental disorders, has been linked to gut microbial imbalances (Yang et al. 2022). The microbiome is also a site of metabolism of nutrients, drugs, and other xenobiotics, and likely alters the disposition of PCBs (Zimmermann et al. 2019). Like other environmental pollutants (Yi et al. 2024; Kou et al. 2025; Lin et al. 2025), PCBs and PCB metabolites can also affect the gut microbiome (Choi et al. 2013; Petriello et al. 2018; Rude et al. 2019; Lim et al. 2020; Agarwal et al. 2023). A study found significant negative correlations between gut microbiome alpha diversity and levels of two PCB congeners, PCB28 and PCB52 (Zhang et al. 2024). These studies focus exclusively on changes in gut bacteria following PCB exposure. The effects of PCB exposure on other microbial kingdoms, such as fungi, remain unexplored.

The interplay between PCBs and gut microbiota may contribute to the toxicity or detoxification of PCBs during gestation and lactation. This interplay between PCBs and the gut microbiome can modulate host or microbiome metabolism and the immune system, ultimately leading to adverse effects on maternal and fetal health. Nonetheless, critical knowledge gaps remain regarding the impact of PCBs and their metabolites on gut microbiota dynamics and functionality during gestation and lactation. To address this knowledge gap, intestinal content samples from a broader animal study focused on the developmental neurotoxicity of the MARBLES mix were used to analyze the microbiome and quantify PCBs and PCB metabolites in post-weaning dams (Sethi et al. 2019; Keil Stietz et al. 2021). This study aimed to characterize, for the first time, associations between individual PCB congeners and metabolites and gut microbiome disruption in post-weaning mouse dams following exposure to the environmentally relevant MARBLES mix. These results provide a mechanistic framework for future studies investigating how PCB-induced alterations of the gut microbiome may contribute to adverse maternal and neonatal outcomes.

## Materials and methods

### Chemicals and PCB standards

The nomenclature of PCBs in this study is according to the U.S. EPA’s classification of 209 PCBs (EPA 2023). Hydroxylated PCB (OH-PCB) abbreviations are adapted from the system introduced by Maervoet et al. (2004), in which the first numeral indicates the hydroxyl group’s position on the biphenyl ring, followed by the parent congener number. Comprehensive details regarding the sources of analytical standards and reagents are available in Dean et al. (2025) and further outlined in the supplementary information.

### Characterization of the MARBLES mix

The 12 PCB congeners in the MARBLES mixture were prepared as reported earlier (Sethi et al. 2019). The MARBLES mix consists of PCB28 (48.2%), PCB11 (24.3%), PCB118 (4.9%), PCB101 (4.5%), PCB52 (4.5%), PCB153 (3.1%), PCB180 (2.8%), PCB149 (2.1%), PCB138 (1.7%), PCB84 (1.5%), PCB135 (1.3%), and PCB95 (1.2%). PCBs (20 mg/mL) were dissolved in organic peanut oil (Spectrum Organic Products, LLC, Melville, NY), which was subsequently diluted into organic peanut butter (Trader Joe's, Monrovia, CA) and orally administered to mice as described below.

### Animals and exposure

All animal procedures complied with the NIH Guide for the Care and Use of Laboratory Animals and received approval from the University of Wisconsin-Madison Animal Care and Use Committee (protocol ID: V006099). For this study, C57BL/6 J mice were obtained from Jackson Labs (strain #000664, Bar Harbor, ME). Mice were housed in clear plastic cages on corn cob bedding. They were maintained on a 12 h light and dark cycle at 22 ± 2 °C. Rodent chow (2019 Teklad Diet, Teklad, Indianapolis, IN) and water were provided ad libitum. All mice were acclimated to the vivarium for 1 week before the dosing began. No adverse effects were observed during the study. Female adult nulliparous mice were dosed daily with the MARBLES mix for two weeks prior to mating and during gestation and lactation (Sethi et al. 2019; Keil Stietz et al. 2021). For the animals used in this study, the total duration of dosing was 63 ± 4 days. Female mice were exposed daily to 0, 0.1, 1, or 6 mg of MARBLES mix per kg of body weight per day, corresponding to the sham, low, medium, and high exposure groups. The PCBs were administered using peanut butter mixtures that contained 0 (vehicle only: peanut oil and peanut butter), 0.025, 0.25, or 1.5 mg MARBLES mix/g peanut butter. These concentrations were designed so that each group of mice could consume approximately the same amount of peanut butter while receiving the intended dose of PCBs. No significant differences in peanut butter consumption were observed among the exposure groups (Table 1), confirming the consistent administration of the desired PCB doses. This exposure paradigm has been extensively used in prior studies and does not produce overt maternal toxicity, pregnancy complications, or gross abnormalities, while resulting in measurable tissue PCB burdens. Although behavioral outcomes were not assessed in the present study, this dosing regimen has been shown to induce alterations in behavior, gut microbiome, and voiding function in offspring and exposed dams in previous investigations (Rude et al. 2019; Keil Stietz et al. 2021; Kennedy et al. 2022; Lavery et al. 2023; Ridlon et al. 2025).

**Table 1:** Dam descriptive statistics of body mass and age at collection, and peanut butter and PCB consumption

	[PCB] mg/kg/d				F (DFn, DFd)	*P* value
	0	0.1	1	6		
N	5	6	5	5		
Mean age (days) of dams at collection	109	110	109	108	F (3, 17) = 0.1455	*P* = 0.9312
Std. deviation	5	6	4	5		
Mean body mass (g) of dams at collection	24.5	25.4	25.4	25.2	F (3, 17) = 0.4134	*P* = 0.7455
Std. deviation	1.5	1.0	1.7	1.6		
Mean total amount (mg) of peanut butter consumed	5.9	6.1	5.7	5.9	F (3, 17) = 0.6965	*P* = 0.5669
Std. deviation	0.5	0.3	0.4	0.7		
Mean total amount (mg) of PCB consumed		0.15	1.4	8.8	W = 459.5 (2.000, 5.352)	*P* < 0.0001 *0.1 vs 1, *0.1 vs 6; *p* = 0.0002 *1 vs 6
Std. deviation		0.01	0.1	1.0		

Descriptive statistics of dams used in this study, *p* values < 0.05 were considered significant as assessed by one-way ANOVA or Welch's one-way ANOVA followed by Dunnett's multiple comparisons test

To refine and reduce the number of animals, they were generated as part of a larger study to produce offspring weaned at postnatal day (P) 21, as described above. All dams used in this study (*n* = 5, 6, 5, and 5 for the sham, low, medium, and high exposure groups, respectively) were 109 ± 5 days old at the time of euthanasia, with no significant differences among exposure groups (Table 1). Similarly, the body mass was comparable across all exposure groups (Table 1). Before euthanasia, dams underwent voiding physiology assays as described (Lavery et al. 2023). Following anesthetized cystometry (Lavery et al. 2023), mice were euthanized via CO_2_ asphyxiation, and the intestine was removed and cut into sections. No gross abnormalities were viewed at the time of necropsy across exposure groups. Intestinal content from the small intestine and cecum was squeezed onto aluminum foil, immediately weighed, and snap-frozen in dry ice. Samples were stored at −80 °C.

### Microbiome analysis

Microbiome analyses of the gut bacteria and fungi were conducted following protocols outlined in previous studies (Shahi et al. 2019; Yadav et al. 2022). DNA was extracted using the DNeasy PowerLyzer PowerSoil Kit (Qiagen, Germantown, MD) and stored at − 80 °C until use. DNA concentration was measured with a Nanodrop spectrophotometer (Thermo Scientific, Waltham, MA). The V3–V4 hypervariable region of the 16S rRNA gene for bacteria and the internal transcribed spacer (ITS) region of the 18S rRNA gene for fungi were amplified and sequenced using the Illumina MiSeq platform (Illumina, San Diego, CA, USA) with paired-end reads. Resulting FASTQ files were processed in R using the dada2 pipeline (Callahan et al. 2016; Shahi et al. 2019), merging paired reads and removing chimeric sequences to obtain amplicon sequence variants (ASVs). Bacterial and fungal ASV tables were analyzed separately with MicrobiomeAnalyst (Dhariwal et al. 2017; Chong et al. 2020). ASVs were retained if they met the following criteria: minimum count of 200, present in at least 20% of samples, and within the interquartile range after removing 10% of ASVs with the lowest abundance. Relative log expression (RLE) transformation was applied before statistical analyses. Alpha diversity, representing within-group diversity, was calculated at the “feature level” using the Shannon index (Kers and Saccenti 2022). Beta diversity, reflecting differences between groups, was assessed by Principal Coordinates Analysis (PCoA) based on Jensen–Shannon Divergence (Chen et al. 2021; Kers and Saccenti 2022). Group-associated taxa were identified using random forest classification (5,000 trees, seven predictors, randomness enabled) to determine ASV importance, while differential abundance testing was performed using Linear Discriminant Analysis Effect Size (LEfSe) (Segata et al. 2011).

### Topic modeling

Topic modeling was conducted using R studio (V. 4.3.1) (R Core Team 2021; Fitzjerrells et al. 2024). First, a phyloseq object (version 1.44.0) containing both microbiome sequence data and associated metadata was created (McMurdie and Holmes 2013). The dataset initially consisted of 283 ASVs; however, filtering based on a prevalence threshold of < 1 × 10⁻^5^ reduced this to 25 ASVs for analysis. For all low, medium, and high exposure groups, the optimal topic number was estimated using multiple evaluation metrics (Cao et al. 2009; Arun et al. 2010) via the ldatuning package (version 1.0.2) (Nikita and Chaney 2020). The “VEM” algorithm for variational inference was applied to construct topics. Latent Dirichlet allocation was then conducted using the topicmodels package (Grün and Hornik 2011; Grün et al. 2024) to finalize topic assignments for each group. From the resulting topic structure, a document–term matrix was created and converted into a new phyloseq object to assess how samples were distributed across topics. Differences in topic prevalence between exposure groups were evaluated using the LinDA method implemented in the MicrobiomeStat package (Zhou et al. 2022; Xhang et al. 2024), with parameters set to feature.dat.type = “count”, is.winsor = FALSE, and p.adj.method = “BH”. Topics were considered significantly associated with an exposure group if the p-value was ≤ 0.05 and the false discovery rate (FDR) was ≤ 0.25.

### Analysis of PCBs and OH-PCBs in intestinal contents

#### Extraction of PCBs and OH-PCBs

The PCB extraction protocol has been described previously (Dean et al. 2025). Briefly, PCB and OH-PCB in the fecal contents were extracted using liquid–liquid extraction based on their partition coefficients in organic and aqueous phases. Concentrated extracts went through a sulfuric acid clean up to remove lipids and were analyzed by GC–MS/MS. A detailed extraction method is available in the supplementary information.

#### GC–MS/MS analysis of PCBs and OH-PCBs

Quantification of PCBs and OH-PCBs, after methylation with diazomethane, was performed using a triple quadrupole gas chromatography–mass spectrometry system (GC–MS/MS) as reported earlier (Dean et al. 2025). The instrumental setup is described in the supplementary information, with specific precursor ions, product ions, and collision energies detailed in Table S2. Analyte levels were calculated using the internal standard method and are adjusted for surrogate recoveries.

To ensure analytical accuracy, precision, and reproducibility, quality assurance and quality control (QA/QC) measures included method blanks, ongoing precision and recovery (OPR) standards, surrogate standards, and internal standards. The detailed QA/QC measures were described in the supplementary information. The results of OPR recoveries, surrogate standard recoveries, method detection limits (MDLs), and limit of detection (LODs) for each PCB congener are summarized in Tables S3-S5.

### Semi-targeted analysis for PCB metabolites in intestinal contents

#### Extraction of PCB metabolites

Following previously established methods, the extraction and semi-targeted analysis of PCB metabolites from intestinal contents were performed using liquid chromatography-high-resolution mass spectrometry (LC-HRMS) (Li et al. 2021). Briefly, intestinal contents (33 ± 4 mg, *n* = 28) were extracted using acetonitrile with 1% formic acid, following spiking with F-tagged 3-fluoro-4-chlorobiphenyl-4'-ol (3-F,4'-OH-PCB3) and its sulfate conjugate as surrogate standards. Phase separation was facilitated by salt addition and centrifugation, and the organic layer was purified using HybridSPE cartridges. Extracts were dried, reconstituted, and normalized with perfluorooctane sulfonic acid (PFOS) before final centrifugation and storage at −80 °C for LC-HRMS analysis. A detailed extraction protocol has been described in the supplementary information.

#### LC-HRMS analysis of PCB metabolites

Polar PCB metabolites in extracts from the intestinal contents were analyzed at the University of Iowa HRMS Facility with a Q-Exactive Orbitrap mass spectrometer (ThermoFisher Scientific, Waltham, MA, USA) coupled with a Vanquish Flex UHPLC system. All details regarding LC-HRMS instrumental setup were described in the supplementary information.

#### Data processing and metabolite identification

LC-HRMS data files (.raw) were processed using Thermo Xcalibur software, version 4.1. Peaks were extracted with a mass tolerance of 5 ppm, five-digit mass precision, and a smoothing factor of 7. Identification of PCB metabolites was confirmed by evaluating chlorine isotopic patterns, as described in prior studies (Li et al. 2021). The relative levels of the metabolite are reported based on peak areas normalized to PFOS. Only metabolites present in at least three samples per exposure group were included in the final analysis.

#### QA/QC for LC-HRMS analyses

To monitor background contamination and instrument carryover, solvent and method blanks were analyzed alongside all samples. No PCB metabolites were found in any blank samples. Surrogate standards, 3-F,4'-OH-PCB3 and its sulfate, were added to all samples to evaluate extraction efficiency and ensure analytical reproducibility. Recovery rates were 44 ± 17% (*n* = 29) for 3-F,4'-OH-PCB3 and 92 ± 40% (*n* = 29) for its sulfate conjugate.

### Network analysis for PCB metabolites and bacterial abundance

To investigate the relationships between PCB/metabolite concentrations and microbial taxa in intestinal samples, a multi-omics network analysis was performed using xMWAS (version 1.0) (Uppal et al. 2018). This platform integrates multivariate datasets through partial least-squares (PLS) regression. xMWAS ranks network nodes based on eigenvector centrality to highlight key associations (Blondel et al. 2008; Cao et al. 2008). The correlation threshold was set to > 0.4 or 0.5 and *p* < 0.05 for Student's t-test. Cytoscape (version 1.0), a widely used tool for biological network representation (Shannon et al. 2003; Otasek et al. 2019), was used to visualize and annotate the networks.

### Statistical analysis

Data are presented as mean ± standard deviation. Descriptive statistics (body mass, age at collection, and peanut butter/PCB intake) were analyzed in Prism v10.0.3 (RRID:SCR_002798) with the dam as the statistical unit (Table 1). For microbes identified by random forest analysis (mean decrease accuracy > 0.01), log-fold changes were calculated, and group differences were assessed using Mann–Whitney U tests. Normality and variance were evaluated using Shapiro–Wilk and Kolmogorov–Smirnov tests, and Bartlett’s test, respectively, and group comparisons were conducted using one-way or Welch’s ANOVA with Dunnett’s post hoc test, as appropriate. Statistical significance was set at *p* < 0.05. PCB and OH-PCB congener profile similarity was assessed by calculating the similarity coefficient cos θ as described in the Supplementary Information (Davis 2002). The value of cos θ = 1 indicates identical profiles, while cos θ = 0 signifies complete dissimilarity.

## Results and discussion

### Microbiome

We found no changes in alpha diversity (Shannon Diversity) in the bacterial community (Fig. S1A) or fungal community (Fig. S1B) of PCB-exposed post-weaning dams. The analysis of beta diversity (Jensen-Shannon Divergence) showed that bacterial and fungal populations did not differ between exposure groups following PCB exposure (Fig. S1C-D). These results align with a prior study examining the microbiome of mouse pups whose mothers were exposed to the MARBLES mix via their diet (Rude et al. 2019). In the earlier study, no differences in beta diversity were noted among the exposure groups.

Although LefSe analysis did not reveal any significant changes in microbial abundance across exposure groups, random forest analysis identified specific bacteria and fungi that were important for distinguishing exposure groups (Fig. S2). However, none of these taxa reached statistical significance after correction for multiple comparisons. Notably, the largest exposure-associated fold changes were observed within the fungal community rather than the bacterial community. Specifically, the relative abundance of fungi within the phylum Basidiomycota, including the species *Malassezia restricta*, consistently increased across all MARBLES exposure doses compared with sham-exposed animals. *Malassezia restricta* has been linked to intestinal inflammation and is reported to be elevated in individuals with inflammatory bowel diseases, particularly Crohn’s disease (Zhang et al. 2022), suggesting that PCB-associated shifts in the mycobiome may have functional relevance. Due to limited understanding of the fungal, viral, and other non-bacterial components of the gut microbiome in relation to environmental toxicant exposure, further studies are necessary to assess whether exposure to PCBs has a selective impact on these components and whether any resulting changes contribute to adverse health effects.

Because conventional diversity and differential abundance analyses did not reveal statistically significant changes in bacterial and fungal abundance, we next applied topic modeling to investigate whether PCB exposure was associated with more subtle, co-occurring bacterial community structures that traditional metrics may not capture (Fitzjerrells et al. 2024). The cosine similarity analysis revealed six topics, each based on ASVs present in the low-dose and sham groups (Fig. S3A), none of which were significant community types. This finding suggests that low-dose PCB exposure did not alter the bacterial community structure at the topic level in a manner that is distinguishable from controls.

**Fig. 1:**
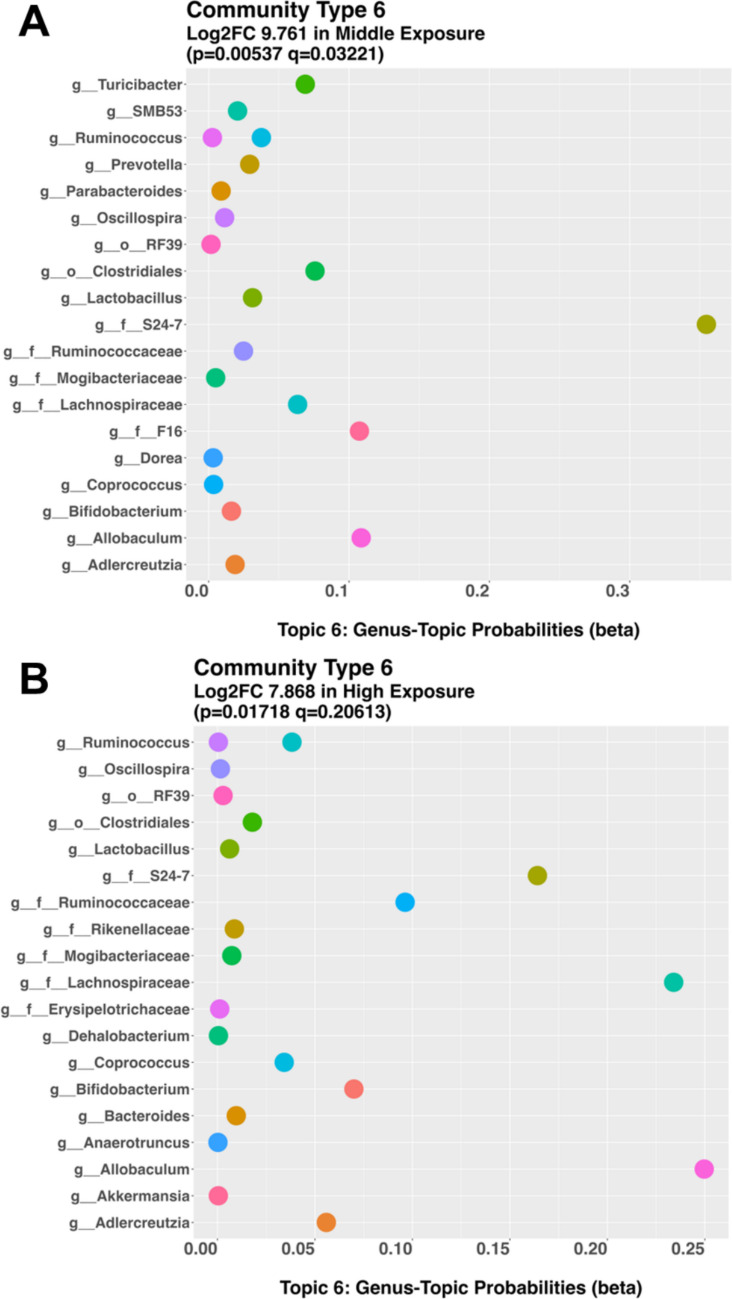
Topic modeling analysis identified a distinct microbial community that was significantly more prevalent in the control group compared to post-weaning mouse dams exposed to the MARBLES mixture at (**A**) medium and (**B**) high doses. In the resulting plots, the x-axis indicates the probability of bacterial taxa being assigned to this community, while the y-axis lists the top 20 taxa with the highest assignment probabilities. Genus (g), Order (o) and Family (f) are labeled to help distinguish which level of bacteria is assigned to each community

Of the six topics created based on ASVs present between the medium dose and sham, the sixth topic was an important community type, characterized by high cosine values (> 0.80; *p* = 0.005; q = 0.032) (Fig. S3B). Nineteen bacteria are found within this community (Fig. 1A). This community has a higher probability of being assigned to the medium-dose group than to the sham group, indicating that medium-dose PCB exposure is associated with a distinct co-occurring bacterial assemblage that is not detected by traditional diversity metrics.

Additionally, of the 12 topics created based on ASVs present between the high-dose and sham groups, the sixth topic was a significant community type (*p* = 0.017; q = 0.21) (Fig. S3C). The 19 bacteria identified in this community are shown in Fig. 1B. This community has a higher probability of being assigned to the high-dose group than to the sham group, suggesting that high PCB exposures result in dose-dependent formation of distinct bacterial community structures.

Thirteen bacterial taxa were identified in the two topics that distinguished the medium and high-dose group from the sham group (Fig. S3D-P), including two orders (clostridiales, RF39), four families (*ruminococcaceae*, *mogibacteriaceae*, *s24-7*, and *lachnospiraceae*), and seven genera (*oscillospira*, *ruminococcus*, *lactobacillus*, *coprococcus*, *bifidobacterium*, *allobaculum*, and *adlercreutzia*). The identification of these taxa with topic modeling across medium- and high-dose groups indicates that these bacteria may respond to PCB exposure, potentially serving as a stable community signature associated with exposure to the MARLES mix.

None of these 13 taxa were detected in the random forest analysis, highlighting that topic modeling captures community-level patterns that may not be detected by single-taxon or machine-learning–based approaches. The family *lachnospiraceae* was the only taxon identified using topic modeling that was significantly altered based on the Mann–Whitney U test after correction for multiple comparisons (Fig. S3I) when comparing the sham and low exposure groups (*p* = 0.0303), consistent with earlier findings that a low dose of the Fox River PCB mixture increased various *lachnospiraceae* genera in female C57BL/6 J mice (Lim et al. 2020). However, *lachnospiraceae* abundance was not significantly different based on the Mann–Whitney U test when comparing the medium- or high-dose groups to the sham group. Together, these findings suggest that exposure to the MARBLES mix may influence gut microbial communities in a dose-dependent manner, with community-level restructuring emerging at medium and high doses despite minimal changes in overall diversity or in the abundance of individual taxa.

### Targeted PCBs and OH-PCBs analysis by GC–MS/MS

#### PCB profiles and levels

**Fig. 2:**
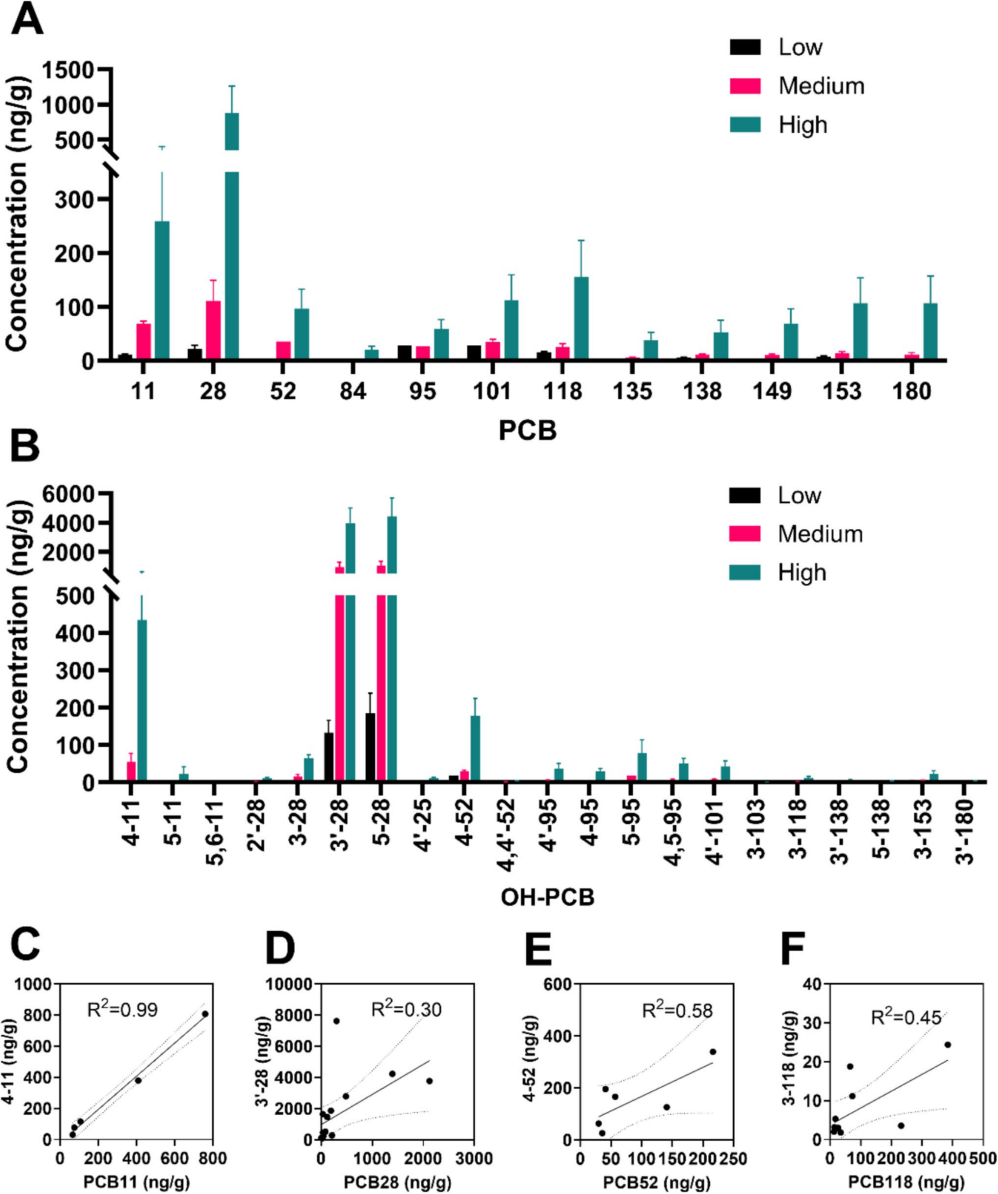
Targeted analysis has identified PCBs and their hydroxylated metabolites in intestinal contents in post-weaning dam mice after MARBLES exposure. (**A**) PCB and (**B**) OH-PCB concentrations (ng/g) in intestinal contents are displayed in bar graphs (mean ± S.E., *n* = 6 for L, and *n* = 5 for M and H). (**C**-**F**) Simple linear regression models were applied to predict the correlation of selected PCBs and their corresponding OH-PCB metabolites. Each plot includes the best-fit regression line (solid) along with 95% confidence intervals (dashed). The coefficient of determination (R^2^) is reported to indicate the strength of association

**Fig. 3:**
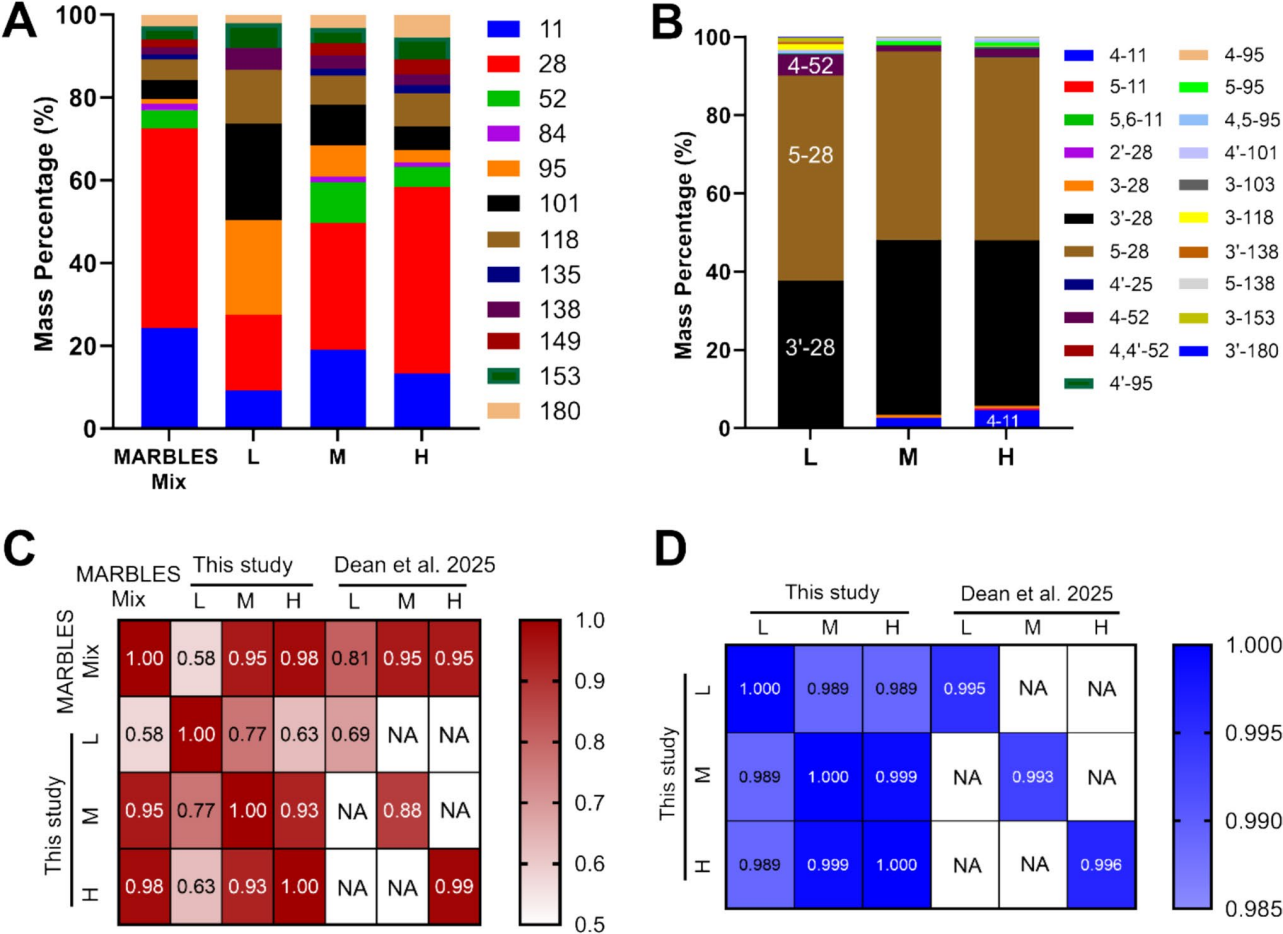
The distribution of PCB and hydroxylated-PCB in the intestinal contents in post-weaning dam mice after MARBLES exposure. Stacked bar graphs have illustrated (**A**) PCB profile and (**B**) OH-PCB profile (in mass percentage %) in the intestinal contents in post-weaning dam mice after MARBLES exposure. To assess profile resemblance, the cosine similarity coefficients (cos θ) were calculated and visualized as a color-coded in a heatmap to compare the profiles of (**C**) PCB and (**D**) OH-PCB. Both profiles were compared to feces from a sister study in which non-pregnant mice were exposed to MARBLES mix for the same duration (Dean et al. 2025). Additionally, the PCB profile was compared to the original MARBLES mixture composition in A and C. NA, not applicable

All twelve PCB congeners included in the MARBLES mixture were identified in the intestinal contents of animals exposed to the medium- and high-dose treatments. However, in the low-dose group, only eight of the 12 congeners exceeded the detection limit. Overall, PCB levels increased in a dose-dependent manner (Fig. 2A). PCB11 and PCB28 made up nearly 60% of the total PCBs detected in the high-dose group. This percentage decreased to 50% in the medium-dose group and further dropped to 27% in the low-dose group (Fig. 3A). In contrast, the combined percentage of PCB95 and PCB101 increased from 9% in the high-dose group to 17% in the medium-dose group, and then to 46% in the low-dose group (Fig. 3A). The PCB mass profile in the high-dose group was identical to that in the MARBLES mix (cos θ = 0.98), whereas the low-dose group showed lower similarity (cos θ = 0.58) compared to the MARBLES mix (Fig. 3C). These findings suggest that PCB dose influences intestinal PCB profiles, possibly through nonlinear absorption at lower exposure levels.

The PCB profiles in the intestinal contents were similar to those in a prior study examining PCB levels in feces from MARBLES-exposed non-pregnant female mice (Fig. 3C) (Dean et al. 2025). However, the PCB levels observed in our study (Table S6) were lower than those found in non-pregnant mice. This difference is due to the partial transfer of PCBs to the fetus during pregnancy and their subsequent transfer to the offspring during lactation (Keil Stietz et al. 2021). PCBs have been reported with detectable levels in the placenta, cord blood, and breast milk across various population studies (Schecter et al. 1998; Suzuki et al. 2005; Han et al. 2022), raising significant concerns about their potential impact on neonatal brain development.

#### OH-PCB profiles and levels

OH-PCBs detection frequencies and levels (Table S6) in the intestinal contents were also dose-dependent. Twenty-one OH-PCBs were detected in the high-dose group, while 19 were found in the medium-dose group and 12 in the low-dose group (Fig. 2B). Two metabolites of PCB28, specifically 5–28 and 3'−28, accounted for approximately 90% of all the OH-PCBs, followed by 4–52 and 4–11 (Fig. 3B). The OH-PCBs mass profiles were identical across all exposure groups, with a cos θ ≥ 0.98 (Fig. 3D). Trace amounts of OH-PCBs metabolites derived from PCB95, PCB101, PCB118, PCB138, PCB153, and PCB180 were discovered, while no OH-PCBs metabolites from PCB84, PCB135, and PCB149 were observed (Fig. 3B). These measurements were based on targeted screening using commercially available or laboratory-synthesized standards, which means other possible OH-PCB metabolites may not have been captured.

Notably, the profiles of OH-PCBs were nearly identical to those observed in our earlier study using MARBLES mix-exposed non-pregnant mice (Dean et al. 2025) (Fig. 3D). The levels of OH-PCBs in this study were significantly lower than those observed in non-pregnant mice, with trends similar to those of PCBs. These findings suggest that the relative formation and excretion of OH-PCBs and their conjugates into the gastrointestinal tract are independent of the degree of chlorination across different PCB doses; however, the absolute metabolite levels are modulated by pregnancy and lactation.

Epidemiological studies have leveraged correlations between the levels of individual PCB congeners and OH-PCBs to infer metabolic relationships and identify which OH-PCBs originate from which PCB congener in humans (Park et al. 2007). Building on this approach, we examined correlations between PCB levels and corresponding OH-PCB metabolites in gastrointestinal contents using simple linear regression models for selected congeners (Fig. 2C-F). Strong positive correlations were observed for several PCB-OH-PCB pairs, particularly PCB11 and its hydroxylated metabolite. However, these relationships did not follow a consistent trend with increasing degree of chlorination, suggesting that factors beyond chlorination may influence the levels of OH-PCBs and their conjugates in the gastrointestinal tract.

### Semi-targeted PCB metabolite analysis by LC-HRMS

**Fig. 4:**
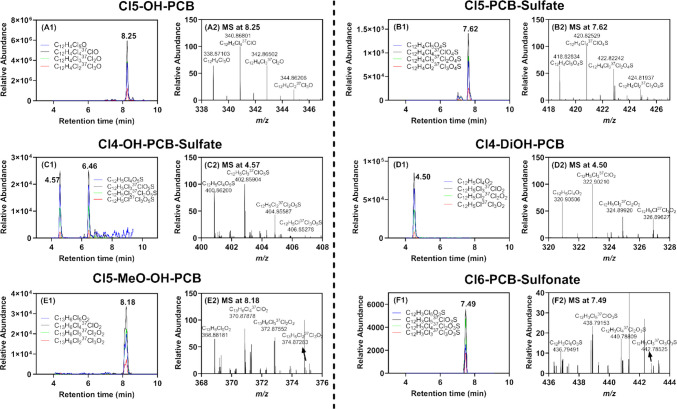
The chromatogram (displayed in A1 to F1) and the corresponding molecular ion mass spectra (shown in A2 to F2) for selected metabolites via the semi-targeted measurement. The metabolites include penta-chlorinated (Cl_5_) OH-PCBs (A1-A2), penta-chlorinated (Cl_5_) PCB sulfates (B1-B2), tetra-chlorinated (Cl_4_) OH-PCB sulfates (C1-C2), tetra-chlorinated (Cl_4_) diOH-PCBs (D1-D2), penta-chlorinated (Cl_5_) MeO-OH-PCBs (E1-E2), and hexa-chlorinated (Cl_6_) PCB sulfonates (F1, F2). In **A1-F1**, chromatograms show the four most abundant isotopic peaks, each represented in a distinct color. Panels **A2-F2** present the corresponding mass spectra, highlighting the measured accurate mass-to-charge ratios (m/z), which closely match the theoretical molecular ion masses and exhibit the expected chlorine isotopic distribution. More information can be found in Table S7

The polar metabolites of PCBs were analyzed using semi-targeted LC-HRMS. Six distinct classes of metabolites were identified, including hydroxylated PCB (OH-PCBs, C_12_H_9-n_Cl_n_O, *n* = 2–7; 16 total analytes), PCB sulfates (C_12_H_9-n_Cl_n_SO_4_, *n* = 1, 3–6; 7 total analytes), hydroxylated PCB sulfates (OH-PCB sulfates, C_12_H_9-n_Cl_n_SO_5_, *n* = 1–2, 4–6; 9 total analytes), dihydroxylated (diOH-PCBs, C_12_H_5_Cl_4_O_2_; 1 analyte), methoxylated OH-PCBs (MeO-OH-PCBs, C_13_H_11-n_Cl_n_O_2_, *n* = 5–6; 2 total analytes), and PCB sulfonates (C_12_H_9-n_Cl_n_SO_3_, *n* = 1 and 6; 2 total analytes) (see Table S7 for more details of each class and analyte). For selected chromatograms of metabolites in each class and the corresponding mass spectrometric data, see Fig. 4.

**Fig. 5:**
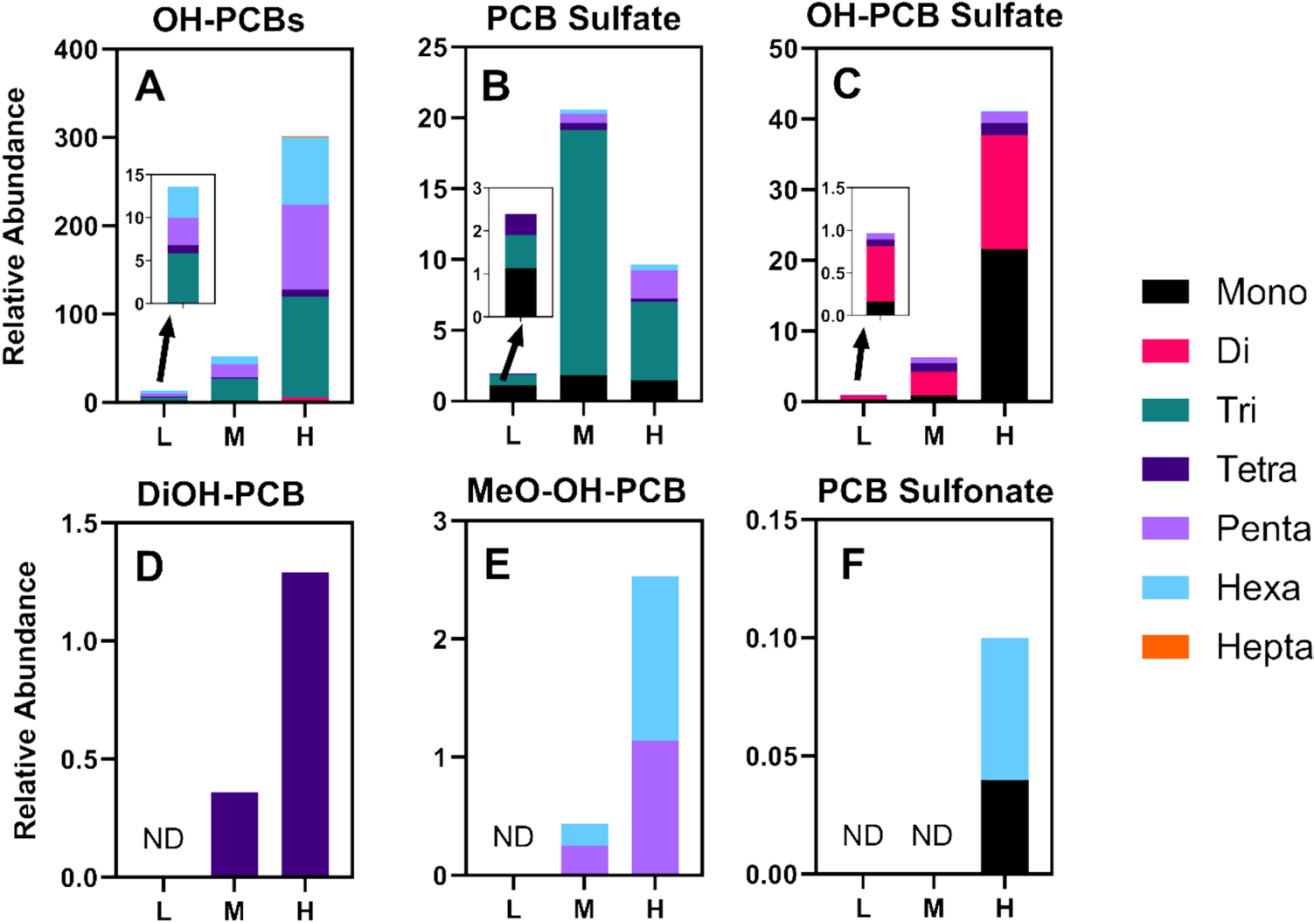
Semi-targeted analysis of PCB metabolites using LC-HRMS revealed multiple homologs of (**A**) hydroxylated (OH)-PCBs, (**B**) PCB sulfates, (**C**) hydroxylated (OH)-PCB sulfates, (**D**) dihydroxylated (diOH)-PCBs, (**E**) methoxylated (MeO)-OH-PCBs, and (**F**) PCB sulfonates in the intestinal contents of post-weaning female dam mice after MARBLES exposure. The graph displays the relative abundance of each homolog that are adjusted by the internal standard PFOS. ND: not detected

The concentrations of analytes in each class typically increased in a dose-dependent relationship. Tri-chlorinated (tri-Cl) PCB sulfates, which are likely derived from PCB28, are a notable exception (Fig. 5). The levels of these polar PCB metabolites in the high-dose group were lower than those in the medium-dose group. PCB sulfates are primarily formed through the conjugation of OH-PCB via sulfotransferases (SULT) in the liver (Grimm et al. 2015). However, once these sulfates enter the gut via biliary secretion, they are subjected to hydrolysis to OH-PCB by the gut microbiome through the action of sulfatase enzymes (Grimm et al. 2015). The observed decrease in tri-Cl PCB sulfate levels at higher doses could result from several dose-dependent metabolic alterations. These include inhibition or saturation of SULT enzymes, depletion of the sulfate donor 3'-phosphoadenosyl-5'-phosphosulfate (PAPS), or inhibition of microbial sulfatase activity (Grimm et al. 2015).

**Fig. 6:**
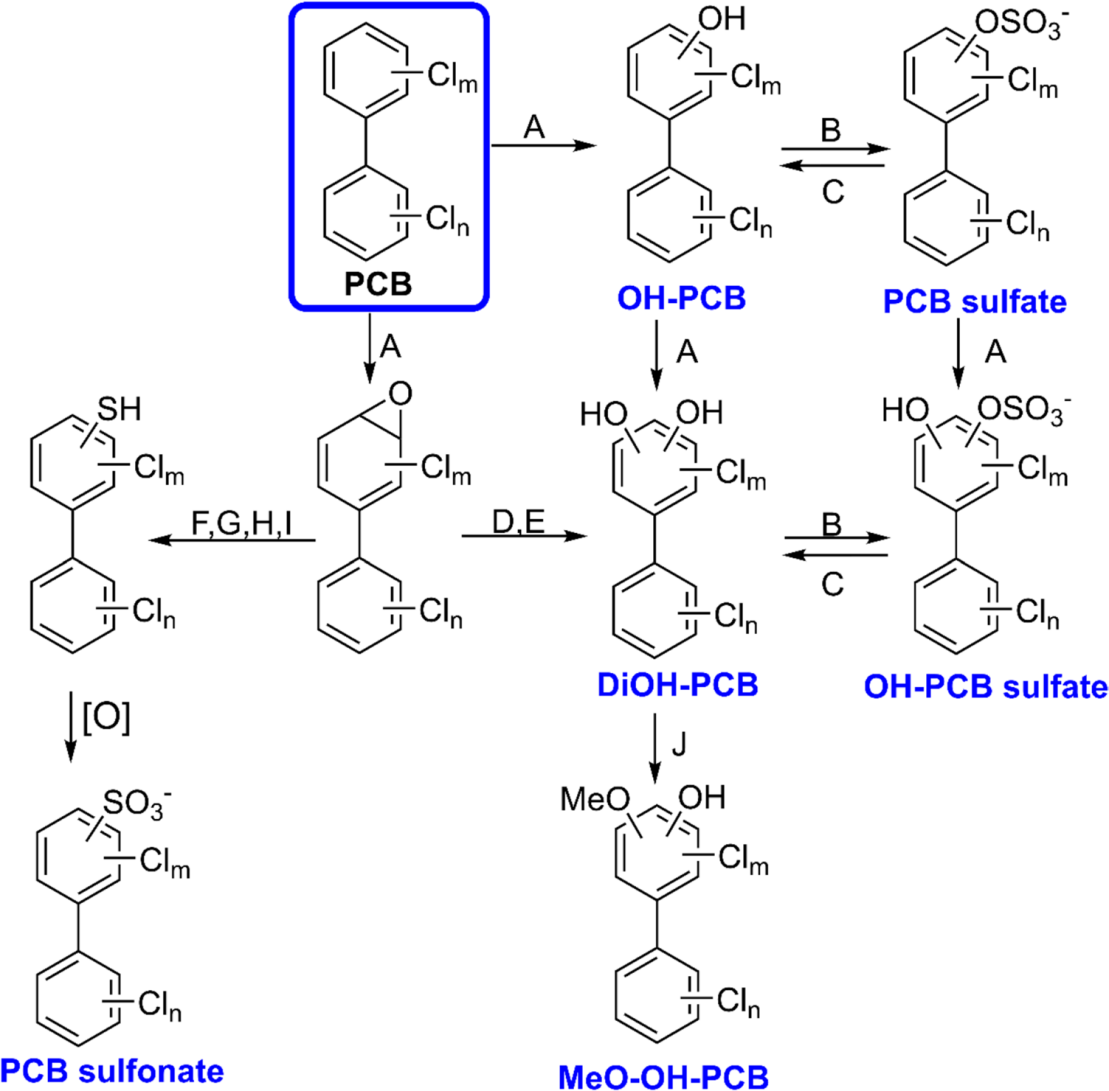
Analysis of intestinal contents from post-weaning female mice after exposure to the MARBLES mixture revealed six distinct classes of PCB metabolites. Due to the lack of authentic standards, the position of the substituents on the biphenyl ring is uncertain. The enzymes likely involved in each metabolism step are (**A**) cytochrome P450 (CYP), (**B**) sulfotransferase (SULT), (**C**) sulfatase, (**D**) epoxide hydrolase (EH), (**E**) dihydrodiol dehydrogenase (DDH), (**F**) glutathione-S-transferase (GST), (**G**) gamma-glutamyl transpeptidase (GGT), (**H**) cysteinylglycinase, (**I**) cysteine-S-conjugate β-lyase, and (**J**) catechol-O-methyltransferase (COMT)

OH-PCBs, phase 1 metabolites formed by cytochrome P450 enzymes, were the metabolite class with the highest levels detected in the intestinal contents. The OH-PCBs accounted for 65%−85% of the total metabolites, with tri- to hexa-chlorinated OH-PCBs being predominant (Fig. 5A). PCB sulfates (3%−26% of total metabolites), followed by OH-PCB sulfates (6%−12% of total metabolites), oxidation products of PCB sulfates (Grimm et al. 2015), were other abundant polar PCB metabolites. DiOH-PCBs, MeO-OH-PCBs, and PCB sulfonate metabolites were minor PCB metabolites detected in the intestinal content. Interestingly, the PCB metabolite profiles were nearly identical to those observed in our earlier study, which exposed non-pregnant mice to the MARBLES mix (Dean et al. 2025). Unlike our previous study, MeO-PCB sulfates were not observed in this study. Based on our results, we propose the PCB metabolic pathway in post-weaning mouse dams, as shown in Fig. 6.

### Integration of PCB, PCB metabolite, and gut microbiome data

**Fig. 7:**
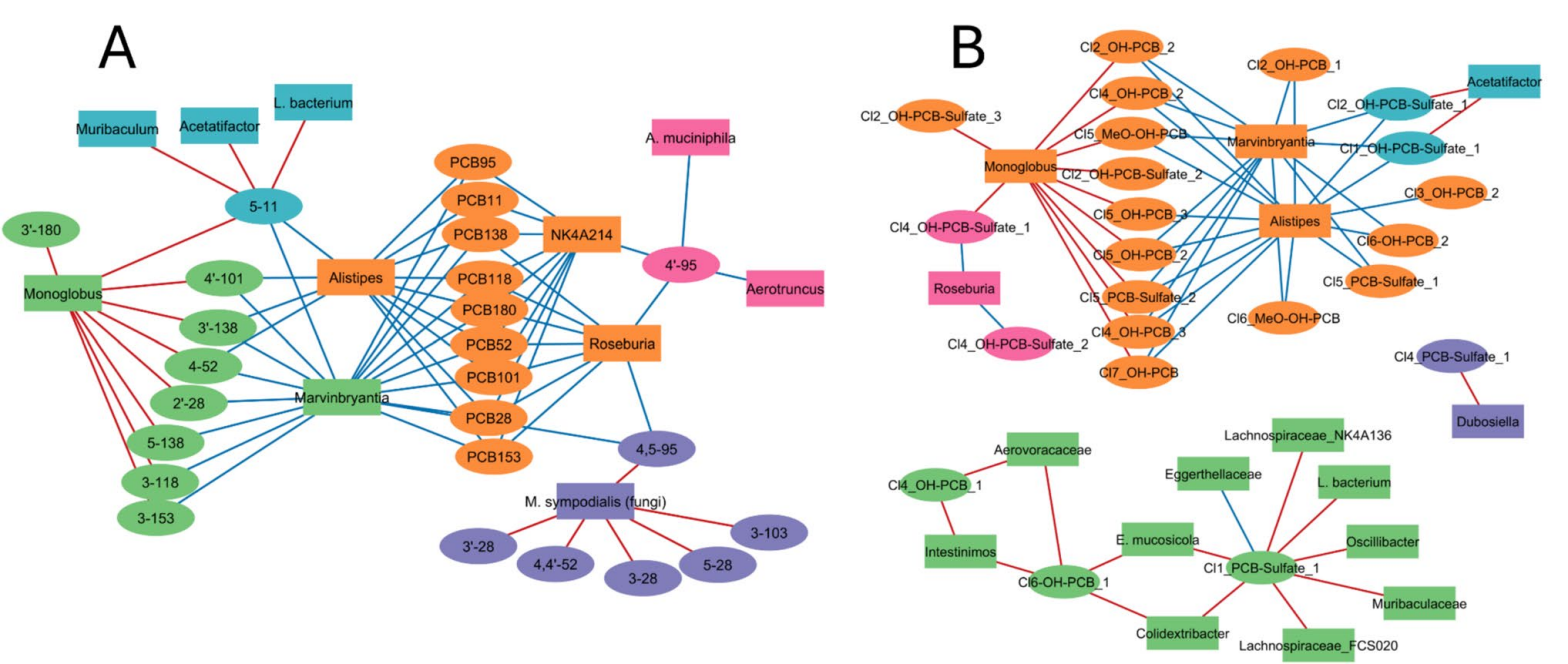
Interactive network analyses using xMWAS identified connections and clusters between (**A**) PCB and OH-PCB levels and gut microbiota abundance, and (**B**) semi-targeted PCB metabolites levels and gut microbiota abundance. Analyses were conducted using an absolute correlation coefficient threshold of > 0.4 for Panel A and > 0.5 for Panel B, with statistical significance determined by Student’s t-test (*p* < 0.05). Nodes sharing the same color represent distinct clusters. Node shapes distinguish entity types: ovals denote PCBs or their metabolites, while rectangles represent bacterial or fungal taxa. Edge colors indicate the direction of correlation: red for positive and blue for negative associations

Network analysis using xMWAS was employed to separately integrate quantified PCB and OH-PCB concentrations determined by targeted GC–MS/MS analysis and the relative abundances of polar PCB metabolites measured by LC-HRMS, along with microbial abundances (Fig. 7). These analyses were performed independently because GC–MS/MS provides absolute, quantitative concentration data. In contrast, LC-HRMS produces relative abundance data for polar metabolites that are not directly comparable on the same scale. The goal of each network analysis was to determine whether specific PCBs or PCB metabolites consistently correlated with changes in microbial abundances in post-weaning mouse dams after PCB exposure. By combining chemical and microbiome data, these network analyses offer a system-level view of how PCB exposure and metabolism may intersect with gut microbial ecology, beyond what traditional diversity or differential abundance analyses can reveal.

#### Integration of PCB and OH-PCB metabolite levels with gut microbiome data

Five clusters were identified in the network of PCB and OH-PCB levels in relation to microbial abundances (Fig. 7A; R = 0.4, *p* < 0.05). The network comprised 25 PCBs and OH-PCBs, as well as 11 microbes, with 70 correlations (18 positive and 52 negative). As with all correlation-based network analyses, these associations do not imply causality but instead identify candidate chemical-microbial relationships for future mechanistic testing. One cluster included nine PCBs (PCB11, PCB28, PCB52, PCB95, PCB101, PCB118, PCB138, PCB153, and PCB180) negatively correlated with three bacterial genera (*Alistipes*, *NK4A214*, and *Roseburia*), suggesting that higher burdens of these congeners may be associated with reduced abundance of commensal taxa linked to gut homeostasis and short-chain fatty acid metabolism.

A second cluster included eight OH-PCBs (4'−101, 3'−138, 4–52, 2'−28, 5–138, 3–118, 3–153, and 3'−180), which were positively correlated with the genus *Monoglobus* and negatively correlated with the genus *Marvinbryantia*. These observations raise the possibility that microbial community composition may respond to the intestinal accumulation of these OH-PCBs. Negative correlations between 4'−95 and the genus *Aerotruncus* and species *A. muciniphila* were included in a third cluster. In contrast, cluster four included a positive correlation between the fungal species *M. sympodialis* and six OH-PCBs (4,5–95, 3'−28, 4,4'−52, 3–28, 5–28, and 3–103). The latter observation suggests that fungal taxa may also be responsive to PCB metabolites present in the gastrointestinal tract, highlighting a potential role for the mycobiome in PCB–host interactions. The fifth cluster included positive correlations between OH-PCB 5–11 and two bacterial genera (*Muribaculum and Acetatifactor*) and the bacterial species *L. bacterium*. The positive correlations observed in the network analysis may reflect microbial tolerance to or adaptation to PCBs or their metabolites, whereas negative correlations may indicate sensitivity to PCBs and their metabolites or indirect suppression of specific taxa via PCB-induced alterations to the gut environment.

#### Integration of relative PCB metabolite levels with gut microbiome data

Five clusters, containing 23 PCB metabolites and 16 microbes, were identified in the network of polar PCB metabolites with microbial abundances (Fig. 7B; R = 0.5, *p* < 0.05), with 58 correlations (27 positive and 31 negative). In one cluster, three genera (*Marvinbryantia*, *Monoglobus*, and *Alistipes*) were correlated with 15 PCB metabolites, including nine OH-PCBs, two PCB sulfates, two OH-PCB sulfates, and two MeO-OH-PCB metabolites. Interestingly, *Monoglobus* was positively associated with various PCB metabolites while *Marvinbryantia* and *Alistipes* were negatively correlated. The second cluster included six genera (*Eggerthellaceae*, *Muribaculaceae*, *Oscillibacter*, *Colidextribacter*, *Aerovoracaceae*, and *Intestinimos*), four species (*E. mucosicola*, *L. NK4A136*, *L. bacterium*, and *L. FCS020*), and three PCB metabolites (two OH-PCBs and one PCB sulfate). *Eggerthellaceae* abundance was negatively correlated, while all remaining bacteria in the second cluster were positively correlated with PCB metabolite levels. In the third and fourth clusters, the genus *Acetatifactor* was positively correlated, and *Roseburia* was negatively correlated with two OH-PCB sulfates. The genus *Dubosiella* was positively correlated with a PCB sulfate in the fifth cluster. The opposing associations within these clusters suggest that some taxa may be enriched in environments with higher metabolite levels. In contrast, other taxa may be suppressed, reflecting differential sensitivity to PCB-derived compounds or distinct metabolic niches.

Interestingly, five bacterial genera (*Acetatifactor*, *Alistipes*, *Marvinbryantia*, *Monoglobus*, and *Roseburia*) and one species (*L. bacterium*) were identified in both network analyses. *Acetatifactor* was positively correlated with lower-chlorinated parent OH-PCB and OH-PCB sulfate metabolites. *Alistipes*, *Marvinbryantia*, and *Roseburia* were negatively correlated with both high- and low-chlorinated PCBs and PCB metabolites. *Monoglobus* was positively correlated with OH-PCBs and various PCB metabolites. *L. bacterium* was positively correlated with one OH-PCB (5–11) and a mono-chlorinated PCB sulfate. The recurrence of these taxa across independent networks underscores their potential as candidate taxa associated with intestinal PCB and PCB metabolite profiles. Collectively, these findings suggest that PCB exposure is associated with coordinated shifts in a defined subset of gut microbes, identifying potential PCB-microbiome interactions that warrant targeted mechanistic investigation.

## Conclusions

This study provides novel insights into how exposure to an environmentally relevant PCB mixture, the MARBLES mix, intersects with gut microbiome organization in post-weaning mouse dams. We observed minimal changes in overall diversity or in the abundance of individual microbial and fungal taxa; however, topic modeling revealed exposure-associated, dose-dependent shifts in co-occurring bacterial community structures that were not captured by conventional diversity or differential abundance analyses. Specifically, the topic modeling revealed that exposure to the MARBLES mix was associated with restructuring of gut microbial communities that emerged at medium and high doses, suggesting that PCB exposure may subtly reorganize microbial community structure even in the absence of large compositional changes. Although these community-level shifts were modest, their presence at the two higher PCB doses suggests their potential relevance for adverse outcomes during pregnancy and lactation associated with PCB exposure. Network-based analyses provided additional system-level insights into how PCB exposure and PCB metabolism may interact with the gut microbiome by identifying associations of specific PCBs or metabolites with particular taxa. These findings suggest that PCB exposure can cause dose-dependent changes in microbial community organization. The interpretation of these findings needs to account for several limitations, including the modest sample size, cross-sectional sampling at a single post-weaning time point, reliance on amplicon-based sequencing, which limits functional inference, and the correlational nature of network analyses, which precludes causal conclusions. Future studies should characterize causal and temporal relationships among changes in the microbiome, PCB and PCB metabolite profiles, and maternal and neonatal health outcomes in pregnant mice using functional approaches, such as shotgun metagenomics, and mechanistic animal models.

## Supplementary Information

Below is the link to the electronic supplementary material.

ESM 1 (PDF 755 KB)

## Data Availability

The sequencing data generated in this study have been deposited in the Sequence Read Archive (https://www.ncbi.nlm.nih.gov/sra; last accessed June 2025) and are publicly accessible under the BioProject ID PRJNA1272122. All other data reported in this manuscript has been published on Iowa Research Online and can be accessed at 10.25820/data.007799.

## Declarations

### Clinical trial number

This is not applicable.
