# Supplementary material for: The influence of an environmentally relevant polychlorinated biphenyl mixture on the intestinal microbiota in post-weaning mouse dams
Source: Environ Sci Pollut Res Int. 2026 Jan 22;33(4):1399–415. doi: 10.1007/s11356-026-37418-3 (PMC12901190; doi:10.1007/s11356-026-37418-3)
Supplement: Supplementary file 1 — (PDF 755 KB) [file 11356_2026_37418_MOESM1_ESM.pdf]

The influence of an environmentally relevant polychlorinated biphenyl mixture on the intestinal microbiota in post-weaning mouse dams

Hui Wang[1,†], Laura E. Dean[1,†], Xueshu Li[1], Rachel L. Fitzjerrells[2,3], Kai Wang[4], Ashutosh K. Mangalam[2,5], Rachel F. Marek[6], Conner L. Kennedy[7], Monica M. Ridlon[7], Audrey Spiegelhoff[7], Kimberly P. Keil Stietz[7], Hans-Joachim Lehmler[1]*

[1]The University of Iowa Department of Occupational and Environmental Health, Iowa City, IA, United States of America

[2]The University of Iowa Interdisciplinary Graduate Program in Informatics, Iowa City, IA, United States of America

[3]The University of Iowa College of Dentistry, Iowa City, IA, United States of America

[4]The University of Iowa Department of Biostatistics, Iowa City, IA, United States of America

[5]The University of Iowa Department of Pathology, Iowa City, IA, United States of America

[6]The University of Iowa Department of Civil and Environmental Engineering, Iowa City, IA, United States of America

[7]University of Wisconsin-Madison Department of Comparative Biosciences, Madison, WI, United States of America

***Corresponding Author:**

Dr. Hans-Joachim Lehmler
The University of Iowa Department of Occupational and Environmental Health
Iowa City, IA United States of America
Phone: (319) 335-4310
Fax: (319) 335-4290
e-mail: hans-joachim-lehmler@uiowa.edu

[†] Both authors contributed equally to this manuscript.

Table of Contents

Abbreviations	S3
Chemicals and PCB Standards	S4
Extracting PCBs and OH-PCBs from Intestinal Contents	S5
GC-MS/MS Instrumental Setup	S7
Quality Assurance and Quality Control (QA/QC) for Target Analyses	S7
Isolation of PCB Metabolites from Intestinal Contents	S8
LC-HRMS Instrumental Setup	S8
Calculation of the Similarity Coefficient cos θ	S9
**Table S1**. Abbreviations and unique identifiers of the analytical OH-PCB standards used in this study.	S10
**Table S2.** Precursor ions, product ions, and collision energies for each analyte in the GC-MS/MS analysis.	S12
**Table S3.** Ongoing Precision and Recovery (OPR) for selected PCBs and OH-PCBs in method blanks and tissue matrices.	S14
**Table S4.** Recoveries (%) of surrogate standards for PCB and OH-PCB in method blank and feces.	S15
**Table S5.** Method Detection Limits (MDLs) and Limits of Detection (LODs) for PCBs from MARBLES mixture and their possible OH-PCB metabolites.	S16
**Table S6**. The concentration (ng/g) of PCB and OH-PCB in feces.	S17
**Table S7**. Semi-target analysis revealed 6 classes of PCB metabolites.	S18
**Fig. S1.** Microbiome diversity plots show no significant differences within (Alpha Diversity) (A) or between (Beta Diversity) (B) groups. Mycobiome diversity plots also showed no significant difference in Alpha Diversity (C) or Beta Diversity (D).	S20
**Fig. S2.** Random forest analysis indicated various bacteria (A) and fungi (B) important for distinguishing exposure groups.	S21
**Fig. S3.** Two minimization metrics were utilized to identify the ideal topic number in low (A), middle (B), and high (C) exposure groups.	S22

Abbreviations

ANOVA	Analysis of Variance
ASVs	amplicon sequence variants
EPA	Environmental Protection Agency
ESI	electrospray ionization
GC-MS/MS	gas chromatography-tandem mass spectrometry
HRMS	high-resolution mass spectrometry
KEGG	Kyoto Encyclopedia of Genes and Genomes
LC	liquid chromatography
LEfSe	Linear Discriminant Analysis Effect Size
LODs	limit of detection
MARBLES	Markers of Autism Risk in Babies: Learning Early Signs
MDLs	method detection limits
OH-PCBs	hydroxylated polychlorinated biphenyls
OPR	ongoing precision and recovery
PAPS	3'-phosphoadenosyl-5'-phosphosulfate
PCBs	polychlorinated biphenyls
PCoA	Principal Coordinates Analysis
PFOS	perfluorooctane sulfonic acid
QA/QC	quality assurance and quality control
RLE	relative log expression
SULT	sulfotransferases

Chemicals and PCB Standards

The 12 PCBs in the MARBLES mixture for animal exposure were synthesized and authenticated by the University of Iowa Superfund Research Program Synthesis Core as described (Li et al., 2018; Sethi et al., 2019). The analytical standards of PCBs and OH-PCBs for the gas chromatography-tandem mass spectrometry (GC-MS/MS) analysis were purchased from AccuStandard (New Haven, CT, USA) unless otherwise specified. 2,5-Dichlorobiphenyl-4'-ol (4'-9), 3,3'-dichlorobiphenyl-4-ol (4-11), 2,3',4-trichlorobiphenyl-4'-ol (4'-25), 2,2',5,5'-tetrachlorobiphenyl-4-ol (4-52), 2,2',3,4',6-pentachlorobiphenyl-4-ol (4-91), 2,2',3,5',6-pentachlorobiphenyl-4-ol (4-95), and 2,2',3,5',6-pentachlorobiphenyl-5-ol (5-95) were synthesized as previously described (Lehmler and Robertson, 2001; Joshi et al., 2011; Rodriguez et al., 2016; Alam et al., 2018). Methoxy PCBs (MeO-PCBs) were synthesized by the Suzuki coupling reaction between (chlorinated) benzene boronic acids and methoxylated bromochlorobenzenes (Lehmler and Robertson, 2001) and authenticated in previous studies (McLean et al., 1996; Zhai et al., 2011; Zhu et al., 2013; Rodriguez et al., 2016; Li et al., 2018; Dhakal et al., 2020; Saktrakulkla et al., 2020)

A few calibration standards were prepared to identify and quantify PCBs and OH-PCBs. A PCB calibration standard mixture containing all 209 PCB congeners was obtained from AccuStandard. For the analysis of OH-PCBs as their corresponding methylated derivatives, two MeO-PCB calibration standard solutions were employed. The first MeO-PCB standard solution consisted of 72 MeO-PCBs, including 70 mono-MeO-PCBs and 2 di-MeO-PCBs, sourced from AccuStandard or Wellington Laboratories (Guelph, ON, Canada). The second MeO-PCB standard solution contained 52 MeO-PCBs, comprising 28 mono-MeO-PCBs and 24 di-MeO-PCBs. Details of the two sets of MeO-PCB standards were published earlier (Dean et al., 2025).

In addition, an OH-PCB standard solution was prepared, which included 4-11, 4'-25, 4-52, 4-95, 5-95, 2,2',4,4',5,5'-hexachlorobiphenyl-3-ol (3-153), and 2,2',3,4',5,5'-hexachlorobiphenyl-4-ol (4-146), all at a concentration of 100 ng/mL in methanol.

For the suspect-screening liquid chromatography-high resolution mass spectrometry (LC-HRMS) analyses, two F-tagged standards were utilized (Dhakal et al., 2012). In brief, 3-fluoro-4-chlorobiphenyl-4'-ol (3-F,4'-OH-PCB3) was synthesized from Suzuki coupling of 1-bromo-3-fluoro-4-chlorobenzene and 4-methoxyphenylboronic acid (Joshi et al., 2011), followed by demethylation using BBr3 (McLean et al., 1996). The resulting 3-F,4'-OH-PCB3 was then converted to sulfuric acid mono-(4'-chloro-3'-fluoro-biphenyl-4-yl) ester, ammonium salt (3-F,4'-PCB3 sulfate), via the corresponding 2,2,2-trichloroethyl-protected sulfate diester (Li et al., 2010).

All organic solvents (pesticide grade), potassium chloride (KCl), sodium chloride (NaCl), hydrochloric acid (HCl), sulfuric acid, formic acid, magnesium sulfate ($MgSO_4$), and sodium sulfate (Na_2SO_4) were obtained from Fisher Scientific (Fair Lawn, NJ, USA). Sodium acetate was acquired from RPI Corp (Mount Prospect, IL, USA). Tetrabutylammonium hydrogen sulfate (TBA) was purchased from J.T. Baker (Phillipsburg, NJ, USA).

Extracting PCBs and OH-PCBs from Intestinal Contents

Fecal contents (64±16 mg, n=28) were aliquoted and homogenized in 3 mL sodium acetate buffer (0.2 M, pH 5.0) using a TissueRuptor homogenizer (Qiagen, Hilden, Germany) in medium-sized glass tubes. Method blanks were processed alongside each extraction batch by adding 50 µL of Milli-Q water. To each sample, 60 ng of 3-fluro-4-chlorobiphenyl-4'-sulfate (3-F,4'-PCB3 sulfate) was added as a surrogate standard for PCB sulfate quantification. Enzymatic hydrolysis was initiated by adding 50 µL of sulfatase type H-2 from *Helix pomatia* (Sigma-Aldrich,

Burlington, MA, USA), followed by overnight incubation at 37°C in a shaking water bath to cleave sulfate and glucuronide conjugates. Subsequently, enzyme denaturation was achieved by heating samples at 110°C for 10 minutes.

Multiple surrogate standards (SS) were added to samples to monitor extraction recoveries. SS for PCBs is a mixture containing PCB15 and PCB117 (10 ng each dissolved in isooctane) and for OH-PCBs is a mixture including 4'-9, 4-91, and 4'-159 (10 ng each dissolved in methanol). The extraction proceeded with the addition of 1 mL of 1% (w/w) potassium chloride (KCl), 4 mL of 2-propanol, 1 mL of 6 M hydrochloric acid, and 4 mL of a 1:1 (v/v) mixture of hexane and methyl tert-butyl ether (MTBE). Samples were rotated for 5 minutes and centrifuged at 1,811 g-force for 5 minutes for phase separation. The upper organic layer was transferred to a clean vial containing 4 mL of 1% KCl. The lower aqueous phase was re-extracted with 3 mL of hexane, and the organic layers were combined with the previous organic phase. The mixture was inverted for 5 minutes and centrifuged at 1,811 g-force for 5 minutes. After mixing for 5 minutes and centrifugation at 1,811 g-force, the final organic phase was collected and concentrated to ~0.5 mL under a gentle nitrogen stream.

We derivatized the OH-PCBs in the samples to methoxylated PCBs (MeO-PCBs) to facilitate GC-MS/MS analysis. This process was achieved by adding 3-5 drops of methanol (methyl source) and about 0.12 mmol of diazomethane (Black, 1983) at 4°C overnight. The excessive diazomethane was removed under nitrogen. For further purification, 2 mL of 2-propanol and 2 mL of 0.1 M tetrabutylammonium hydrogen sulfate (TBA) were added. The extracts then underwent solid-phase extraction (SPE) cleanup in a cartridge filled with acidified silica gel (sulfuric acid: silica gel = 1: 5, w/w), and PCBs and metabolites were eluted with 14 mL of dichloromethane (DCM). The eluents were concentrated to near dryness, and 3 mL of hexane was

added to reconstitute the sample, which was then concentrated to a final volume of 50 μL. Internal standards (d-PCB30 and PCB204, 10 ng each in hexane) were added and extracts were transferred to a GC vial and ready for GC-MS/MS analysis.

GC-MS/MS Instrumental Setup

Quantification of PCBs and OH-PCBs, after methylation with diazomethane, was performed using a triple quadrupole gas chromatography-mass spectrometry system (GC-MS/MS), consisting of an Agilent 7890B GC, 7000D Triple Quad detector, and 7693 autosampler (Agilent Technologies, Santa Clara, CA, USA) (Dean et al., 2025). The GC was operated with a SPB-Octyl capillary column (30 m length, 0.25 mm inner diameter, 0.25 µm film thickness; 50% n-octyl/50% methyl siloxane). The carrier gas was helium, with a flow rate of 0.8 mL/min. The collision gas was nitrogen. The temperature program of the inlet was 45°C (held for 0.06 min), ramping rapidly to 325°C at 600°C/min under 5 psi pressure. The GC oven temperature was as follows: initial hold at 45°C for 2 min, increase to 75°C at 100°C/min (held for 5 min), then to 150°C at 15°C/min (held for 1 min), and finally to 280°C at 2.5°C/min with a final hold of 5 min. The electron ionization source was maintained at 230°C, and the transfer line was held at 280°C.

Quality Assurance and Quality Control (QA/QC) for Target Analyses

To ensure analytical accuracy, precision, and reproducibility, hexane solvent blanks, method blanks, and ongoing precision and recovery (OPR) standards were included throughout sample processing. Concentrations of PCBs and OH-PCBs were corrected based on the recovery rates of their corresponding surrogate standards. The method detection limits (MDLs) was determined based on the formula from EPA: $MDL = mean_{blank} + t_{(0.01, n-1)} \times SD_{blank}$, where $mean_{blank}$ is the mean concentration in the method blanks, $t_{(0.01, n-1)}$ represents the 99th percentile from a Student's distribution with n–1 degrees of freedom, and SD_{blank} is the standard deviation of the

method blanks (EPA, 2016). The limit of detection (LODs) were similarly estimated using control tissue data: LOD = $mean_{control} + t_{(0.01, n-1)} \times SD_{control}$, where $mean_{control}$ and $SD_{control}$ represent the average concentration and standard deviation, respectively, from control samples.

Isolation of PCB Metabolites from Intestinal Contents

33 ± 4 mg of intestinal contents (n = 28) were weighed and placed into glass tubes. Surrogate standards 3-F,4'-OH-PCB3 and its sulfate conjugate (10 ng each) were spiked into each sample. The extraction was initiated by adding 4 mL of acetonitrile containing 1% formic acid (v/v), and samples were vortexed thoroughly. To facilitate phase separation, 200 mg of NaCl and 800 mg of $MgSO_4$ were added, followed by vigorous mixing. Samples were centrifuged at 1,811 g-force for 5 minutes, and the organic layer was passed through HybridSPE cartridges (Sigma-Aldrich, St. Louis, MO, USA) pre-packed with 3 g of a 1:1 (w/w) blend of $MgSO_4$ and Na_2SO_4. The eluates were dried using a Savant SpeedVac SPD concentrator (Thermo Scientific, Waltham, MA, USA) at 35°C. The dried extracts were reconstituted in 300 µL of acetonitrile and centrifuged at 1,811 g-force for 5 minutes. Supernatants were transferred to microcentrifuge tubes and evaporated at 35°C. Final reconstitution was performed using 200 µL of a 1:1 (v/v) water-acetonitrile mixture. Perfluorooctanesulfonic acid potassium salt (PFOS, 10 ng) was added as an internal standard for volume correction. Samples were stored at -20°C for a minimum of 30 minutes, then centrifuged at 16,000 × g-force for 10 minutes at 4°C. The resulting supernatants were transferred to LC vials with inserts and stored at -80°C until analysis.

LC-HRMS Instrumental Setup

Polar PCB metabolites in extracts from the intestinal contents were analyzed at the University of Iowa HRMS Facility with a Q-Exactive Orbitrap mass spectrometer (ThermoFisher

Scientific, Waltham, MA, USA) coupled with a Vanquish Flex UHPLC system. Chromatographic separation was achieved using an Acquity UPLC-C18 column (2.1 mm I.D., 1.7 µm particle size, 100 mm length; Waters, Milford, MA, USA). Water containing 10 mM ammonium formate and 0.3% (v/v) of ammonium hydroxide was used as mobile phase A. Mobile phase B consisted of methanol-acetonitrile (1:1, v/v) with 10 mM ammonium formate with 0.3% (v/v) of ammonium hydroxide. A flow rate of 0.2 mL/min was used for the separation of the PCB metabolites. The LC system was operated with the following gradient program: initial conditions of 30% B held for 1 minute, followed by a linear increase to 99% B, held for 3 minutes, and then returned to 30% B, with a 4-minute hold before the next injection. Each injection volume was 2 µL.

The Orbitrap mass spectrometer was operated in negative electrospray ionization (ESI) mode, with a spray voltage of 2,472 V and a current of 18.2 µA. Sheath and auxiliary gas flows were set to 48 mL/min and 2 mL/min, respectively. The capillary temperature was maintained at 256°C, and the auxiliary gas heater at 413°C. Full scan data were acquired over an m/z range of 85-1,000, with a resolution of 70,000, a maximum injection time of 200 ms, and an AGC target of 1×10^6.

Calculation of the Similarity Coefficient cos θ

To compare the PCB and OH-PCB profiles of two groups, the similarity coefficient cos θ (ranging from 0-1) was calculated using this formula:

$$\cos\theta = \frac{\sum_{i=1}^{n}(A_i B_i)}{\sqrt{\sum_{i=1}^{n}(A_i^{2})}\sqrt{\sum_{i=1}^{n}(B_i^{2})}}$$

where and are the th components of dataset and , respectively.

Table S1. Abbreviations and unique identifiers of the analytical OH-PCB standards used in this study.

Abbreviation	IUPAC Name	Formula	Isomeric SMILES	InChI	InChIKey	CAS Registry Number	CAS Registry URL	PubChem CID	PubChem Link	DTXSID	Comptox Link
4'-9	4-(2,5-dichlorophenyl)phenol	C12H8Cl2O	C1=CC(=CC=C1C2=C(C=CC(=C2)Cl)Cl)O	InChI=1S/C12H8Cl2O/c13-9-3-6-12(14)11(7-9)8-1-4-10(15)5-2-8/h1-7,15H	BTORSXCJJIWNIS-UHFFFAOYSA-N	53905-28-5	https://commonchemistry.cas.org/detail?cas_rn=53905-28-5	91653	https://pubchem.ncbi.nlm.nih.gov/compound/91653	DTXSID0022351	https://comptox.epa.gov/dashboard/chemical/details/DTXSID0022351
4-91	2,3,5-trichloro-4-(2,4-dichlorophenyl)phenol	C12H5Cl5O	C1=CC(=C(C=C1Cl)Cl)C2=C(C=C(C(=C2Cl)Cl)O)Cl	InChI=1S/C12H5Cl5O/c13-5-1-2-6(7(14)3-5)10-8(15)4-9(18)11(16)12(10)17/h1-4,18H	RSKPYFJMALAIJJ-UHFFFAOYSA-N	NA	NA	101788493	https://pubchem.ncbi.nlm.nih.gov/compound/101788493	NA	NA
4'-159	2,6-dichloro-4-(2,3,4,5-tetrachlorophenyl)phenol	C12H4Cl6O	C1=C(C=C(C(=C1Cl)O)Cl)C2=CC(=C(C(=C2Cl)Cl)Cl)Cl	InChI=1S/C12H4Cl6O/c13-6-3-5(9(16)11(18)10(6)17)4-1-7(14)12(19)8(15)2-4/h1-3,19H	PZAKBNHYWBSZAF-UHFFFAOYSA-N	158076-63-2	https://commonchemistry.cas.org/detail?cas_rn=158076-63-2	178005	https://pubchem.ncbi.nlm.nih.gov/compound/178005	DTXSID70166369	https://comptox.epa.gov/dashboard/DTXSID70166369
4-11	2-chloro-4-(3-chlorophenyl)phenol	C12H8Cl2O	C1=CC(=CC(=C1)Cl)C2=CC(=C(C=C2)O)Cl	InChI=1S/C12H8Cl2O/c13-10-3-1-2-8(6-10)9-4-5-12(15)11(14)7-9/h1-7,15H	JOHAARQQFBMIOV-UHFFFAOYSA-N	53890-78-1	https://commonchemistry.cas.org/detail?cas_rn=53890-78-1	186674	https://pubchem.ncbi.nlm.nih.gov/compound/186674	DTXSID10202159	https://comptox.epa.gov/dashboard/DTXSID10202159
4'-25	2-chloro-4-(2,4-dichlorophenyl)phenol	C12H7Cl3O	C1=CC(=C(C=C1C2=C(C=C(C=C2)Cl)Cl)Cl)O	InChI=1S/C12H7Cl3O/c13-8-2-3-9(10(14)6-8)7-1-4-12(16)11(15)5-7/h1-6,16H	IPQDZKABLRZERH-UHFFFAOYSA-N	358767-68-7	https://commonchemistry.cas.org/detail?cas_rn=358767-68-7	53221454	https://pubchem.ncbi.nlm.nih.gov/compound/53221454	DTXSID50686095	https://comptox.epa.gov/dashboard/chemical/details/DTXSID50686095
4-52	2,5-dichloro-4-(2,5-dichlorophenyl)phenol	C12H6Cl4O	C1=CC(=C(C=C1Cl)C2=CC(=C(C=C2Cl)O)Cl)Cl	InChI=1S/C12H6Cl4O/c13-6-1-2-9(14)7(3-6)8-4-11(16)12(17)5-10(8)15/h1-5,17H	ZKDSNFDCQYBBIU-UHFFFAOYSA-N	51274-68-1	https://commonchemistry.cas.org/detail?cas_rn=51274-68-1	39971	https://pubchem.ncbi.nlm.nih.gov/compound/39971	DTXSID10199272	https://comptox.epa.gov/dashboard/DTXSID10199272

Abbreviation	IUPAC Name	Formula	Isomeric SMILES	InChI	InChIKey	CAS Registry Number	CAS Registry URL	PubChem CID	PubChem Link	DTXSID	Comptox Link
4-95	2,3,5-trichloro-4-(2,5-dichlorophenyl)phenol	C12H5Cl5O	C1=CC(=C(C=C1Cl)C2=C(C=C(C(=C2Cl)Cl)O)Cl)Cl	InChI=1S/C12H5Cl5O/c13-5-1-2-7(14)6(3-5)10-8(15)4-9(18)11(16)12(10)17/h1-4,18H	VLOXUAHEXUHYTO-UHFFFAOYSA-N	NA	NA	102344102	https://pubchem.ncbi.nlm.nih.gov/compound/102344102	NA	NA
5-95	2,4,5-trichloro-3-(2,5-dichlorophenyl)phenol	C12H5Cl5O	C1=CC(=C(C=C1Cl)C2=C(C(=CC(=C2Cl)Cl)O)Cl)Cl	InChI=1S/C12H5Cl5O/c13-5-1-2-7(14)6(3-5)10-11(16)8(15)4-9(18)12(10)17/h1-4,18H	NGZZCCQHHGJRSN-UHFFFAOYSA-N	NA	NA	102344104	https://pubchem.ncbi.nlm.nih.gov/compound/102344104	NA	NA
3-153	2,3,6-trichloro-5-(2,4,5-trichlorophenyl)phenol	C12H4Cl6O	C1=C(C(=CC(=C1Cl)Cl)Cl)C2=CC(=C(C(=C2Cl)O)Cl)Cl	InChI=1S/C12H4Cl6O/c13-6-3-8(15)7(14)1-4(6)5-2-9(16)11(18)12(19)10(5)17/h1-3,19H	ZVJPNYXNOYRCIJ-UHFFFAOYSA-N	54284-55-8	https://commonchemistry.cas.org/detail?cas_rn=54284-55-8	6452977	https://pubchem.ncbi.nlm.nih.gov/compound/6452977	DTXSID90202637	https://comptox.epa.gov/dashboard/DTXSID90202637
4-146	2,3,6-trichloro-4-(2,4,5-trichlorophenyl)phenol	C12H4Cl6O	C1=C(C(=CC(=C1Cl)Cl)Cl)C2=CC(=C(C(=C2Cl)Cl)O)Cl	InChI=1S/C12H4Cl6O/c13-6-3-8(15)7(14)1-4(6)5-2-9(16)12(19)11(18)10(5)17/h1-3,19H	KVRQWFNZIYFJRU-UHFFFAOYSA-N	145413-90-7	https://commonchemistry.cas.org/detail?cas_rn=145413-90-7	3050412	https://pubchem.ncbi.nlm.nih.gov/compound/3050412	DTXSID60163004	https://comptox.epa.gov/dashboard/DTXSID60163004

Table S2. Precursor ions, product ions, and collision energies for each analyte in the GC-MS/MS analysis.

Analyte	Precursor Ion (*m/z*)	Product Ion (*m/z*)	Collision Energy (eV)
3-F,4'-OH-PCB3	237	194	25
4'-9	252	209	20
PCB11	222	152	25
2-11	252	202	25
4-11	252	209	25
5-11	252	222	20
6-11	252	202	25
5,6-11	282	232	25
2,5-11	282	232	25
4,5-11	282	204	25
PCB15	222	152	25
PCB28	256	186	25
d-PCB30	261	191	30
2'-28	286	236	25
3-28	286	243	25
3'-28	286	243	25
5-28	286	243	25
4'-25	286	243	25
PCB52	292	222	25
4-52	322	279	20
4,4'-52	352	337	20
PCB84	326	256	25
4-91	356	313	25
PCB95	326	256	25
4-95	356	313	25
4'-95	356	313	25
5-95	356	313	25
4,5-95	386	343	25
3-103	356	306	25
PCB101	326	256	25
4'-101	356	313	25
6'-101	356	306	25
PCB117	326	256	25
PCB118	326	256	20
3-118	356	313	25
PCB135	360	290	25
PCB138	360	290	25
3'-138	390	347	25
5-138	390	347	25
PCB149	360	290	25

**Analyte**	**Precursor Ion (*m/z*)**	**Product Ion (*m/z*)**	**Collision Energy (eV)**
PCB153	360	290	25
3-153	390	347	25
4-146	390	347	25
4'-159	390	375	15
PCB180	394	323	25
3'-180	424	381	25
PCB204	430	358	25

Table S3. Ongoing Precision and Recovery (OPR) for selected PCBs and OH-PCBs in method blanks and tissue matrices. Values are mean ± SD.

PCB/OH-PCB name	Recovery in method blank (%) (n=2)	Recovery in tissue matrices (%) (n=9)
PCB11	101 ± 2	112 ± 6
PCB15	100 ± 2	119 ± 18
PCB28	101 ± 2	116 ± 10
4'-9	98 ± 0	117 ± 19
PCB52	102 ± 1	124 ± 19
4-11	107 ± 1	123 ± 18
PCB95	99 ± 1	125 ± 23
PCB84	94 ± 1	117 ± 19
4'-25	107 ± 1	116 ± 12
PCB101	100 ± 0	118 ± 14
4-52	104 ± 1	112 ± 9
PCB117	96 ± 0	109 ± 9
PCB135	94 ± 1	98 ± 4
PCB149	96 ± 0	101 ± 3
5-95	105 ± 0	117 ± 16
PCB118	98 ± 1	118 ± 19
4-95	103 ± 1	115 ± 15
4-91	102 ± 1	113 ± 14
PCB153	97 ± 0	99 ± 3
PCB138	96 ± 0	100 ± 2
3-153	101 ± 0	102 ± 2
4-146	101 ± 0	102 ± 2
PCB180	93 ± 0	97 ± 4
4'-159	106 ± 1	104 ± 4
3-F-4'-PCB3 sulfate	93 ± 7	102 ± 14

Table S4. Recoveries (%) of surrogate standards for PCB and OH-PCB in method blank and feces.

Surrogate Standards	Method Blanks (n=4)	Feces (n=27)
PCB15	116 ± 7	130 ± 11
4'-9	120 ± 9	125 ± 14
PCB117	109 ± 4	108 ± 7
4-91	124 ± 2	115 ± 9
4'-159	96 ± 6	102 ± 3
3-F-4'-PCB3 sulfate	104 ± 6	112 ± 7

Table S5. Method Detection Limits (MDLs) and Limits of Detection (LODs) for PCBs from MARBLES mixture and their possible OH-PCB metabolites.

PCB/OH-PCB	MDL[a] (ng)	LOD[b] (ng/g)	PCB/OH-PCB	MDL[a] (ng)	LOD[b] (ng/g)
**PCB11**	0.25	7.24	**4'-95**	<0.001	0.01
**2-11**	0.01	0.19	**5-95**	<0.001	7.69
**4-11**	0.49	18.5	**4,5-95**	0.01	0.12
**5-11**	<0.01	0.03	**3-103[c]**	<0.01	0.04
**6-11**	2.11	34.7	**PCB101**	1.26	27.3
**5,6-11**	<0.01	0.02	**4'-101**	<0.01	<0.01
**2,5-11**	0.11	1.78	**6'-101**	<0.01	0.08
**4,5-11**	0.01	<0.01	**PCB118**	0.45	10.5
**PCB28**	0.12	3.14	**3-118**	0.12	1.70
**2'-28**	0.12	2.24	**PCB135**	0.20	3.45
**3-28**	0.39	6.84	**PCB138**	0.30	5.84
**3'-28**	0.11	4.47	**3'-138**	<0.01	0.02
**5-28**	0.16	6.57	**5-138**	<0.01	0.02
**4'-25[c]**	<0.01	6.40	**PCB149**	0.41	7.59
**PCB52**	1.34	26.0	**PCB153**	0.26	4.97
**4-52**	0.12	9.65	**3-153**	<0.01	0.01
**4,4'-52**	<0.01	0.15	**4-146[c]**	<0.01	0.04
**PCB84**	0.17	3.85	**PCB180**	<0.01	0.13
**PCB95**	1.35	27.5	**3'-180**	<0.01	0.08
**4-95**	<0.01	6.45			

[a] MDL, Method Detection Limits (ng) were calculated using the formula: MDL= $mean_{blank} + t_{0.01, n-1} * SD_{blank}$, where $mean_{blank}$ is the mean of method blanks, $t_{0.01, n-1}$ is Student's t-value for n – 1 degrees of freedom at the 99% confidence level, and SD_{blank} is the standard deviation of the method blanks. N = 8.
[b] LOD, Limit of Detection (ng/g feces) were adjusted by feces mass and were calculated from formula: LOD= $mean_{control} + t_{0.01, n-1} * SD_{control}$, where $mean_{control}$ is the mean of control feces measures, $t_{0.01, n-1}$ is Student's t-value for n – 1 degrees of freedom at the 99% confidence level, and $SD_{control}$ is the standard deviation of the control feces measures. N = 8.
[c] Possible metabolites due to NIH shift.

Table S6. The concentration (ng/g) of PCB and OH-PCB in the intestinal contents.

PCB congener	0.1 mg/kg (n=6)		1 mg/kg (n=5)		6 mg/kg (n=5)	
	Average (f)	SD	Average (f)	SD	Average (f)	SD
**PCB11**	11 (3)	6	69 (2)	6	260 (5)	320
**PCB28**	22 (6)	16	110 (5)	85	880 (5)	850
**PCB52**			36 (1)		97 (5)	80
**PCB84**			4.8 (1)		21 (4)	12
**PCB95**	28(1)		28 (1)		59 (4)	35
**PCB101**	29 (1)		35 (2)	7	110 (5)	100
**PCB118**	15 (2)	8	26 (5)	13	160 (5)	150
**PCB135**			6.0 (2)	1.6	39 (3)	25
**PCB138**	6.5 (2)	3.4	11 (3)	2	52 (5)	51
**PCB149**			11 (2)	3	69 (3)	47
**PCB153**	7.4(2)	4.0	14 (5)	7	107 (5)	106
**PCB180**	2.5 (6)	2.4	12 (5)	8	107 (5)	113
**OH-PCB congener**	**0.1 mg/kg (n=6)**		**1 mg/kg (n=5)**		**6 mg/kg (n=5)**	
	**Average (f)**	**SD**	**Average (f)**	**SD**	**Average (f)**	**SD**
**4-11**			54 (2)	33	430 (3)	350
**5-11**	0.07 (1)		0.04 (2)	0.01	23 (4)	39
**5,6-11**			0.05 (1)		0.15 (2)	0.01
**2'-28**			2.5 (3)	0.2	11 (5)	5
**3-28**			17 (5)	11	64 (5)	23
**3'-28**	130 (6)	80	960 (5)	740	4000 (5)	2300
**5-28**	180 (6)	130	1000 (5)	700	4400 (5)	2900
**4'-25**					12 (2)	3
**4-52**	19 (1)		30 (5)	8	180 (5)	100
**4,4'-52**			1.4 (5)	1.1	3.7 (5)	2.9
**4'-95**	1.3 (4)	0.8	4.5 (5)	5.3	37 (5)	31
**4-95**					29 (5)	19
**5-95**			18(1)		79 (4)	71
**4,5-95**	1.5 (6)	1.3	7.2 (5)	3.6	50 (5)	32
**4'-101**	1.8 (6)	1.3	8.1 (5)	2.2	43 (5)	34
**3-103**			0.2 (4)	0.1	0.78 (5)	0.88
**3-118**	5.3 (1)		2.5 (4)	0.7	12 (5)	9
**3'-138**	1.9 (2)	1.5	1.0 (5)	0.5	5.8 (5)	4.7
**5-138**	0.4 (2)	0.3	1.0 (5)	0.4	3.1 (5)	2.3
**3-153**	3.1 (6)	5.9	5.2 (5)	2.2	23 (5)	17
**3'-180**	0.8 (3)	0.9	0.9 (5)	0.3	4.0 (5)	2.5

Empty values are either not detectable or not available. (f): detection frequency.

Table S7. Semi-target analysis revealed 6 classes of PCB metabolites.

Class No.	Metabolite	RT(RRT)	Normalized Intensity (Detection Frequency)			Formula	[M-H]⁻		m/z, δppm	Confidence Level
			LD N=6	MD N=6	HD N=7		Calculated (Da)	Measured (Da)		
1.1.1	Cl2-OH-PCB	7.73(1.06)	0.12±0.08(3)	0.47±0.59(3)	4.27±5.50(4)	$C_{12}H_7Cl_2O$	236.98794	236.98811	0.70327	1
1.1.2		8.04(1.10)	ND	0.14±0.14(4)	0.74±0.60(5)	$C_{12}H_7Cl_2O$	236.98794	236.98804	0.41714	4
1.1.3		8.28(1.13)	ND	ND	0.89±1.29(5)	$C_{12}H_7Cl_2O$	236.98794	236.98795	0.03416	4
1.2.1	Cl3-OH-PCB	7.84(1.07)	4.29±5.35(6)	17.6±6.3(5)	75.7±43.2(5)	$C_{12}H_6Cl_3O$	270.94897	270.94873	-0.89808	4
1.2.2		8.28(1.13)	1.42±1.12(6)	8.58±3.11(5)	38.3±21.2(5)	$C_{12}H_6Cl_3O$	270.94897	270.94914	0.64441	1
1.3.1	Cl4-OH-PCB	6.90(0.94)	0.56±0.32(5)	0.93±0.63(5)	ND	$C_{12}H_5Cl_4O$	306.90705	306.90725	0.65420	4
1.3.2		7.29(1.00)	0.18±0.11(5)	0.75±0.27(5)	4.93±4.50(5)	$C_{12}H_5Cl_4O$	306.90705	306.90718	0.42203	1
1.3.3		7.47(1.02)	0.09±0.07(4)	0.29±0.27(5)	2.66±3.45(5)	$C_{12}H_5Cl_4O$	306.90705	306.90738	1.08248	4
1.3.4		7.81(1.07)	0.11±0.07(5)	ND	ND	$C_{12}H_5Cl_4O$	306.90705	306.90723	0.59283	4
1.4.1	Cl5-OH-PCB	7.56(1.03)	ND	0.31±0.17(4)	ND	$C_{12}H_4Cl_5O$	340.86808	340.86828	0.58837	4
1.4.2		8.25(1.13)	2.68±2.77(6)	10.9±5.6(5)	88.1±108.5(5)	$C_{12}H_4Cl_5O$	340.86808	340.86966	4.62153	4
1.4.3		8.57(1.17)	0.56±0.90(5)	2.17±0.89(4)	8.14±7.09(4)	$C_{12}H_4Cl_5O$	340.86808	340.86804	-0.10366	4
1.4.4		9.19(1.26)	ND	0.66±0.58(3)	ND	$C_{12}H_4Cl_5O$	340.86808	340.86795	-0.39116	4
1.5.1	Cl6-OH-PCB	7.74(1.06)	1.07±1.10(3)	1.16±0.68(3)	ND	$C_{12}H_3Cl_6O$	374.82910	374.82935	0.65511	4
1.5.2		7.87(1.08)	2.52±3.28(6)	8.54±8.15(5)	76.4±99.28(4)	$C_{12}H_3Cl_6O$	374.82910	374.82926	0.41649	4
1.6.1	Cl7-OH-PCB	8.29(1.13)	ND	0.16±0.10(4)	0.83±0.85(4)	$C_{12}H_2Cl_7O$	408.79013	408.79014	0.03262	4
2.1.1	Cl-PCB Sulfate	5.81(0.79)	1.13±0.43(6)	1.82±0.76(4)	1.48±1.22(4)	$C_{12}H_8ClO_4S$	282.98373	282.98382	0.33178	4
2.2.1	Cl3-PCB Sulfate	6.77(0.92)	0.27±0.14(6)	5.83±7.83(5)	1.95±2.17(5)	$C_{12}H_6Cl_3O_4S$	350.90579	350.90617	1.08472	4
2.2.2		6.93(0.95)	0.50±0.39(6)	11.5±16.7(5)	3.55±3.55(5)	$C_{12}H_6Cl_3O_4S$	350.90579	350.90473	-3.01374	1
2.3.1	Cl4-PCB Sulfate	7.02(0.96)	0.08±0.02(3)	0.50±0.56(5)	0.28±0.30(5)	$C_{12}H_5Cl_4O_4S$	386.86386	386.86425	1.00380	1
2.4.1	Cl5-PCB Sulfate	7.05(0.96)	ND	0.13±0.06(4)	0.26±0.34(4)	$C_{12}H_4Cl_5O_4S$	420.82489	420.82532	1.02180	4
2.4.2		7.62(1.04)	ND	0.53±0.46(5)	1.69±2.65(5)	$C_{12}H_4Cl_5O_4S$	420.82489	420.82542	1.25377	4

Class No.	Metabolite	RT(RRT)	Normalized Intensity (Detection Frequency)			Formula	$[M-H]^-$		m/z, δppm	Confidence Level
			LD N=6	MD N=6	HD N=7		Calculated (Da)	Measured (Da)		
2.5.1	Cl6-PCB Sulfate	7.83(1.07)	ND	0.30±0.08(4)	0.42±0.22(3)	$C_{12}H_3Cl_6O_4S$	454.78592	454.78607	0.33251	4
3.1.1	Cl-PCB Sulfonate	4.73(0.65)	ND	ND	0.04±0.01(4)	$C_{12}H_8ClO_3S$	266.98882	266.98903	0.76782	4
3.2.1	Cl6-PCB Sulfonate	7.49(1.02)	ND	ND	0.06±0.04(3)	$C_{12}H_3Cl_6O_3S$	438.79100	438.79107	0.14813	4
4.1.1	Cl4-DiOH-PCB	4.50(0.61)	ND	0.36±0.22(3)	1.29±0.89(4)	$C_{12}H_5Cl_4O_2$	322.90196	322.90197	0.03441	4
5.1.1	Cl-OH-PCB Sulfate	5.75(0.79)	0.16±0.12(4)	0.88±0.83(5)	21.5±39.6(4)	$C_{12}H_8ClO_5S$	298.97865	298.97885	0.67861	4
5.2.1	Cl2-OH-PCB Sulfate	5.55(0.76)	0.58±0.43(4)	1.71±1.34(5)	13.1±25.7(5)	$C_{12}H_7Cl_2O_5S$	332.93967	332.93956	-0.32696	4
5.2.2		6.17(0.84)	0.07±0.02(3)	1.01±1.04(5)	2.17±2.81(3)	$C_{12}H_7Cl_2O_5S$	332.93967	332.93845	-3.67200	4
5.2.3		6.35(0.87)	ND	0.63±0.56(3)	0.95±1.28(3)	$C_{12}H_7Cl_2O_5S$	332.93967	332.93938	-0.87904	4
5.3.1	Cl4-OH-PCB Sulfate	4.57(0.62)	ND	0.31±0.30(4)	0.59±0.63(3)	$C_{12}H_5Cl_4O_5S$	402.85878	402.85916	0.95360	4
5.3.2		6.46(0.88)	0.08±0.06(3)	0.90±1.22(5)	1.20±1.59(5)	$C_{12}H_5Cl_4O_5S$	402.85878	402.85940	1.52659	4
5.4.1	Cl5-OH-PCB Sulfate	7.28(0.99)	0.08±0.02(5)	0.82±0.80(5)	1.50±1.39(4)	$C_{12}H_4Cl_5O_5S$	436.81981	436.82014	0.75546	4
5.5.1	Cl6-OH-PCB Sulfate	6.64(0.91)	ND	ND	0.13±0.05(3)	$C_{12}H_3Cl_6O_5S$	470.78083	470.78103	0.42483	4
5.5.2		7.11(0.97)	ND	ND	0.07±0.02(3)	$C_{12}H3Cl_6O_5S$	470.78083	470.78050	-0.71158	4
6.1.1	Cl5-MeO-OH-PCB	8.18(1.12)	ND	0.25±0.06(4)	1.14±1.13(4)	$C_{13}H_6Cl_5O_2$	370.87864	370.87878	0.38647	4
6.2.1	Cl6-MeO-OH-PCB	7.85(1.07)	ND	0.19±0.05(4)	1.39±1.28(4)	$C_{13}H_5Cl_6O_2$	404.83967	404.83954	-0.33209	4

Only metabolites that have more than 3 detection frequency are reported. Metabolite levels are shown as mean ± SD (detection frequency). ND: not detected; RT: retention time; RRT: relative retention time, equal to the retention time in relative to internal standard. ppm: parts per million, ppm = (measured -calculated)/calculated* 1,000,000. The accurate mass was calculated/measured based on the most abundant isoform. A confidence level of 4 represents PCB metabolites identified based on the accurate mass and the isotope pattern. A confidence level of 1 indicates the PCB metabolites were identified based on accurate mass, isotope pattern, and authentic standards.

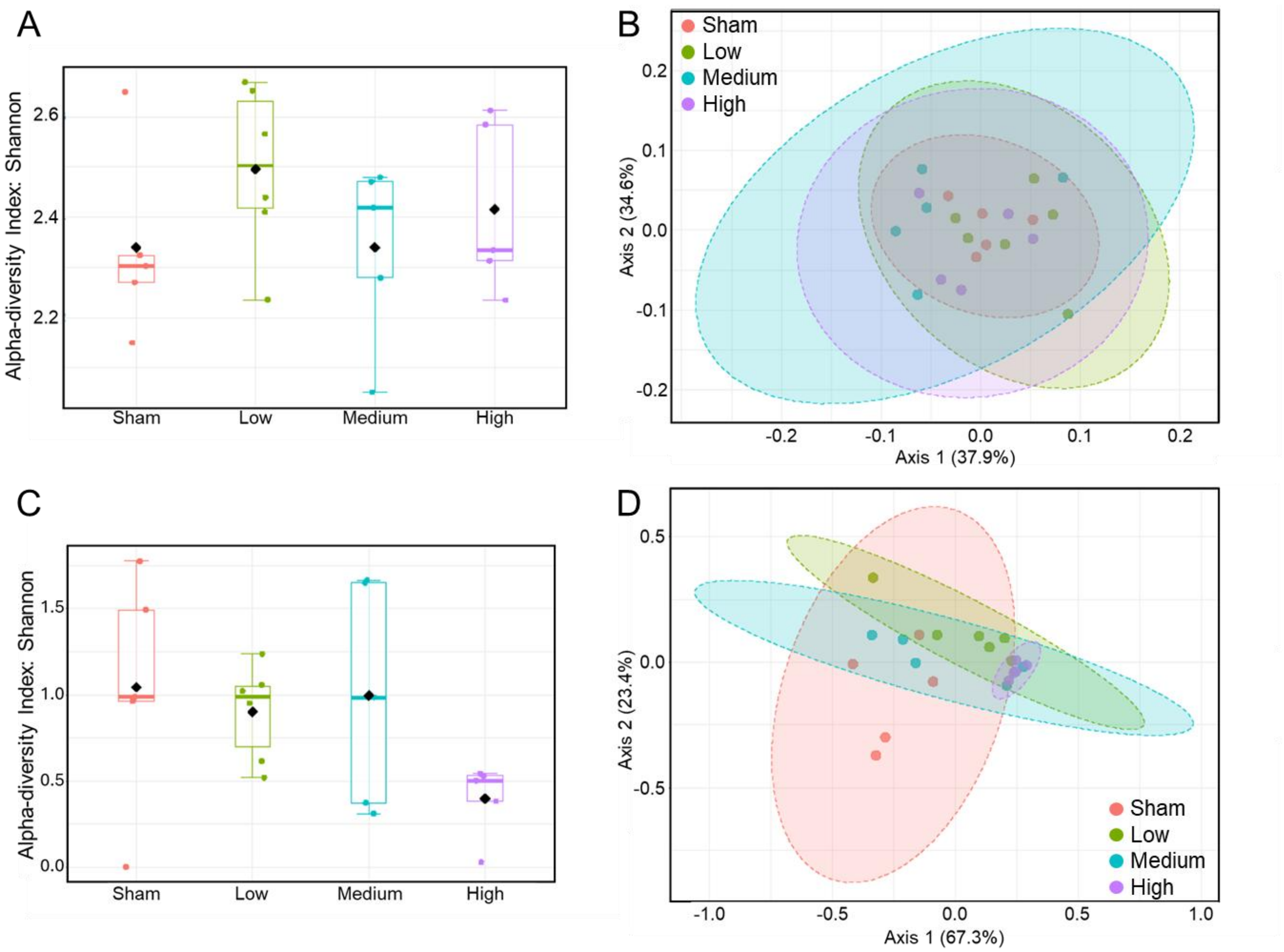

Fig. S1. Microbiome diversity plots show no significant differences within (Alpha Diversity) (A) or between (Beta Diversity) (B) groups. Mycobiome diversity plots also showed no significant difference in Alpha Diversity (C) or Beta Diversity (D). Alpha diversity was measured by Shannon Diversity at the feature level. PCoA and Jensen-Shannon Divergence at the feature level were used to measure beta diversity.

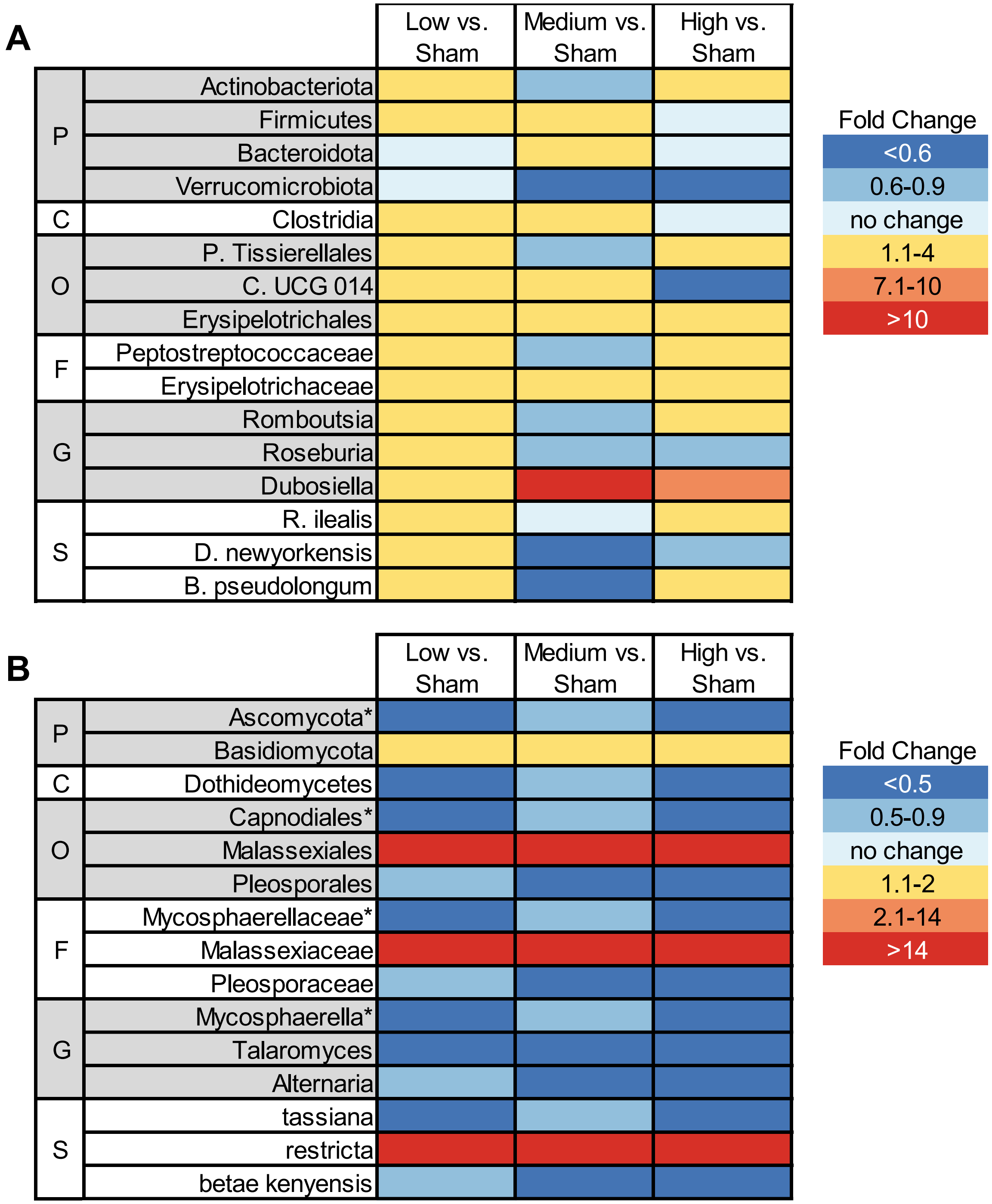

Fig. S2. Random forest analysis indicated various bacteria (A) and fungi (B) important for distinguishing exposure groups.

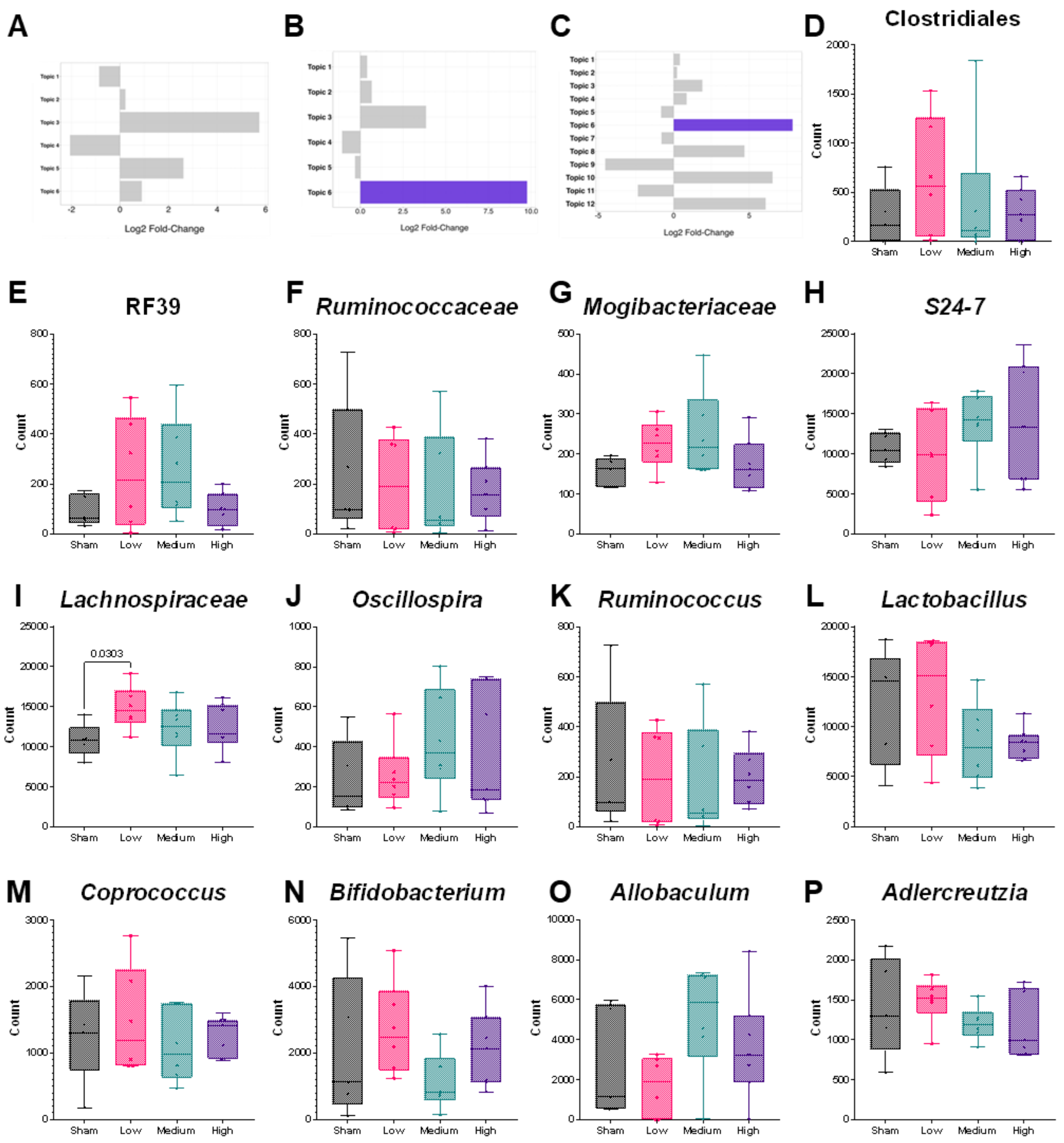

Fig. S3. Two minimization metrics were utilized to identify the ideal topic number in low (A), middle (B), and high (C) exposure groups. The assignment of samples to topics with one topic significantly more often assigned to control mice than medium exposure and control than high exposure mice (B and C, highlighted in purple). There were 13 bacterial taxa that were found in both significant communities (D-P).

References

Alam, S., Carter, G.S., Krager, K.J., Li, X., Lehmler, H.J. and Aykin-Burns, N. (2018). PCB11 Metabolite, 3,3'-Dichlorobiphenyl-4-ol, Exposure Alters the Expression of Genes Governing Fatty Acid Metabolism in the Absence of Functional Sirtuin 3: Examining the Contribution of MnSOD. *Antioxidants (Basel)* 7, https://doi.org/10.3390/antiox7090121.

Black, H. (1983). The preparation and reactions of diazomethane. *Aldrichimica Acta* 16, 3.

Dean, L.E., Wang, H., Li, X., Fitzjerrells, R.L., Valenzuela, A.E., Neier, K., LaSalle, J.M., Mangalam, A., Lein, P.J. and Lehmler, H.J. (2025). Identification of polychlorinated biphenyls (PCBs) and PCB metabolites associated with changes in the gut microbiome of female mice exposed to an environmental PCB mixture. *J Hazard Mater* 489, 137688, https://doi.org/10.1016/j.jhazmat.2025.137688.

Dhakal, K., He, X., Lehmler, H.J., Teesch, L.M., Duffel, M.W. and Robertson, L.W. (2012). Identification of sulfated metabolites of 4-chlorobiphenyl (PCB3) in the serum and urine of male rats. *Chem Res Toxicol* 25, 2796–8204, https://doi.org/10.1021/tx300416v.

Dhakal, R., Li, X., Parkin, S.R. and Lehmler, H.J. (2020). Synthesis of mono- and dimethoxylated polychlorinated biphenyl derivatives starting from fluoroarene derivatives. *Environ Sci Pollut Res Int* 27, 8905–8925, https://doi.org/10.1007/s11356-019-07133-3.

EPA. (2016). Definition and procedure for the determination of the method detection limit, revision 2. *U.S. Environmental Protection Agency*

Joshi, S.N., Vyas, S.M., Duffel, M.W., Parkin, S. and Lehmler, H.J. (2011). Synthesis of Sterically Hindered Polychlorinated Biphenyl Derivatives. *Synthesis (Stuttg)* 7, 1045–1054, https://doi.org/10.1055/s-0030-1258454.

Lehmler, H.J. and Robertson, L.W. (2001). Synthesis of hydroxylated PCB metabolites with the Suzuki-coupling. *Chemosphere* 45, 1119–1127, https://doi.org/10.1016/s0045-6535(01)00052-2.

Li, X., Holland, E.B., Feng, W., Zheng, J., Dong, Y., Pessah, I.N., Duffel, M.W., Robertson, L.W. and Lehmler, H.-J. (2018). Authentication of synthetic environmental contaminants and their (bio)transformation products in toxicology: polychlorinated biphenyls as an example. *Environ Sci Pollut Res* 25, 16508–16521, https://doi.org/10.1007/s11356-017-1162-0.

Li, X., Parkin, S., Duffel, M.W., Robertson, L.W. and Lehmler, H.J. (2010). An efficient approach to sulfate metabolites of polychlorinated biphenyls. *Environ Int* 36, 843–848, https://doi.org/10.1016/j.envint.2009.02.005.

McLean, M.R., Bauer, U., Amaro, A.R. and Robertson, L.W. (1996). Identification of Catechol and Hydroquinone Metabolites of 4-Monochlorobiphenyl. *Chemical Research in Toxicology* 9, 158–164, https://doi.org/10.1021/tx950083a.

Rodriguez, E.A., Li, X., Lehmler, H.-J., Robertson, L.W. and Duffel, M.W. (2016). Sulfation of Lower Chlorinated Polychlorinated Biphenyls Increases Their Affinity for the Major Drug-

Binding Sites of Human Serum Albumin. *Environmental Science & Technology* 50, 5320–5327, https://doi.org/10.1021/acs.est.6b00484.

Saktrakulkla, P., Dhakal, R.C., Lehmler, H.J. and Hornbuckle, K.C. (2020). A semi-target analytical method for quantification of OH-PCBs in environmental samples. *Environ Sci Pollut Res Int* 27, 8859–8871, https://doi.org/10.1007/s11356-019-05775-x.

Sethi, S., Morgan, R.K., Feng, W., Lin, Y., Li, X., Luna, C., Koch, M., Bansal, R., Duffel, M.W., Puschner, B., Zoeller, R.T., Lehmler, H.J., Pessah, I.N. and Lein, P.J. (2019). Comparative analyses of the 12 most abundant PCB congeners detected in human maternal serum for activity at the thyroid hormone receptor and ryanodine receptor. *Environ Sci Technol* 53, 3948–3958, https://doi.org/10.1021/acs.est.9b00535.

Zhai, G., Lehmler, H.-J. and Schnoor, J.L. (2011). New hydroxylated metabolites of 4-monochlorobiphenyl in whole poplar plants. *Chemistry Central Journal* 5, 87, https://doi.org/10.1186/1752-153X-5-87.

Zhu, Y., Mapuskar, K.A., Marek, R.F., Xu, W., Lehmler, H.J., Robertson, L.W., Hornbuckle, K.C., Spitz, D.R. and Aykin-Burns, N. (2013). A new player in environmentally induced oxidative stress: polychlorinated biphenyl congener, 3,3'-dichlorobiphenyl (PCB11). *Toxicol Sci* 136, 39–50, https://doi.org/10.1093/toxsci/kft186.
